# Mice harboring pathobiont-free microbiota do not develop intestinal inflammation that normally results from an innate immune deficiency

**DOI:** 10.1371/journal.pone.0195310

**Published:** 2018-04-04

**Authors:** Benoit Chassaing, Andrew T. Gewirtz

**Affiliations:** 1 Center for Inflammation, Immunity and Infection, Institute for Biomedical Sciences, Georgia State University, Atlanta, GA, United States of America; 2 Neuroscience Institute, Georgia State University, Atlanta, GA, United States of America; "INSERM", FRANCE

## Abstract

**Background:**

Inability to maintain a stable and beneficial microbiota is associated with chronic gut inflammation, which classically manifests as colitis but may more commonly exist as low-grade inflammation that promotes metabolic syndrome. Alterations in microbiota, and associated inflammation, can originate from dysfunction in host proteins that manage the microbiota, such as the flagellin receptor TLR5. That the complete absence of a microbiota (i.e. germfree conditions) eliminates all evidence of inflammation in TLR5-deficient mice demonstrates that this model of gut inflammation is microbiota-dependent. We hypothesize that such microbiota dependency reflects an inability to manage pathobionts, such as Adherent-Invasive *E*. *coli* (AIEC). Herein, we examined the extent to which microbiota mismanagement and associated inflammation in TLR5-deficient mice would manifest in a limited and pathobiont-free microbiota. For this purpose, WT and TLR5-deficient mice were generated and maintained with the 8-member consortium of bacteria referred to as “Altered Schaedler Flora” (ASF). Such ASF animals were subsequently inoculated with AIEC reference strain LF82. Feces were assayed for bacterial loads, fecal lipopolysaccharide and flagellin loads, fecal inflammatory marker lipocalin-2 and microbiota composition.

**Results:**

Relative to similarly maintained WT mice, mice lacking TLR5 (T5KO) did not display low-grade intestinal inflammation nor metabolic syndrome under ASF conditions. Concomitantly, the ASF microbial community was similar between WT and T5KO mice, while inoculation with AIEC strain LF82 resulted in alteration of the ASF community in T5KO mice compared to WT control animals. AIEC LF82 inoculation in ASF T5KO mice resulted in microbiota components having elevated levels of bioactive lipopolysaccharide and flagellin, a modest level of low-grade inflammation and increased adiposity.

**Conclusions:**

In a limited-complexity pathobiont-free microbiota, loss of the flagellin receptor TLR5 does not impact microbiota composition nor its ability to promote inflammation. Addition of AIEC to this ecosystem perturbs microbiota composition, increases levels of lipopolysaccharide and flagellin, but only modestly promotes gut inflammation and adiposity, suggesting that the phenotypes previously associated with loss of this innate immune receptor require disruption of complex microbiota.

## Background

Intestinal inflammation can manifest in a range of forms, including inflammatory bowel disease (IBD) and low-grade inflammation, which is a term that refers to modest elevations in pro-inflammatory gene expression that is not accompanied by classic histopathologically-evident features of inflammation. Such low-grade inflammation is associated with and thought to promote metabolic syndrome, making it important to understand its underlying causes [1]. Host genetics are a major determinant of one’s predisposition to develop intestinal inflammation [2, 3], wherein dysfunction in genes that mediate the host’s ability to detect and respond to gut bacteria increase likelihood of developing chronic inflammation [4–6]. Yet, non-genetic factors, including gut microbiota, are also major determinants of the extent to which such genetic alterations manifest in intestinal inflammation and associated phenotypes. A mouse model that exemplifies, and helped reveal, these concepts are mice deficient in the flagellin receptor toll-like receptor 5 (TLR5). Briefly, depending upon their microbiota composition, which is dictated by their husbandry and vivarium, T5KO mice are prone to developing robust intestinal inflammation, i.e. histopathologically-evident colitis, or low-grade inflammation and associated metabolic disease [4, 5]. Agents that disrupt host-microbiota interactions, including dietary emulsifiers and anti-IL-10 receptor antibodies, increase the incidence and severity of the inflammation exhibited by T5KO mice [7, 8]. In contrast, the complete absence of a microbiota, achieved *via* germfree rederivation, eliminated evidence of gut inflammation and its associated phenotypes, even following treatment with anti-IL-10 receptor antibodies, thus demonstrating the absolute requirement of a microbiota for the gut inflammation associated phenotypes exhibited by these mice [5, 9].

Development of robust gut inflammation in T5KO mice was associated with increased γ-Proteobacteria, particularly in the post-weaning period [10], suggesting a possible role for this class of bacteria, which has been associated with inflammation in a range of disorders [11, 12]. In contrast, development of T5KO metabolic syndrome was associated with a more generalized dysbiosis characterized by a microbiota that expressed higher levels of flagellin and lipopolysaccharide (LPS), thus having a greater inherent capacity to drive pro-inflammatory gene expression [13]. One factor that can promote dysbiosis is adherent-invasive *Escherichia coli* (AIEC) [14], a class of bacteria associated with ileal Crohn’s disease [15–17]. Specifically, oral administration of an AIEC strain during microbiota acquisition by germfree animals resulted in intestinal inflammation in T5KO, but not in WT mice [10]. Such AIEC bacteria are able to colonize WT and T5KO mice only transiently (up to 4 weeks), but yet resulted in lasting alterations in microbiota composition in T5KO mice that correlated with elevated levels of LPS and flagellin [14].

Herein, we sought to better understand how such microbiota alterations might result in colitis and metabolic syndrome. WT and T5KO mice harboring the “Altered Schaedler Flora” (ASF) microbiota were generated. The ASF community is an 8-member consortium of microbes (ASF 356 = *Clostridium sp*.; ASF 360 = *Lactobacillus intestinalis*; ASF 361 = *Lactobacillus murinus*; ASF 457 = *Mucispirillum shaedleri*; ASF 492 = *Eubacterium plexicaudatum*; ASF 500 = *Pseudoflavonifractor sp*.; ASF 502 = *Clostridium sp*.; ASF 519 = *Parabacteroides goldsteinii*) that can stably coexist in the mouse gut and drive development of a seemingly normal intestinal immune system but is devoid of pathogenic or pathobiont bacteria [18]. Yet, it should be noted that while this minimal community is sufficient to improve intestinal immunity and metabolism compared to germfree animals, ASF-colonized mice exhibit an abnormal T cell repertoire [19], lack colonization resistance [20, 21] which might be related to the presence of an unoccupied ecological niche, and an abnormal metabolic capacity [22]. This caveat notwithstanding, we hypothesized that, as a result of the high degree of redundancy in the mucosal immune system, which is reported to be relatively normal in ASF mice [18, 23], T5KO mice would be able to effectively manage this relatively simple microbiota and would not develop intestinal inflammation. Moreover, we hypothesized that addition of AIEC bacteria might disturb this microbiota resulting in chronic inflammation. We herein observed that, when raised under ASF conditions, T5KO animals do not develop intestinal inflammation nor metabolic syndrome. Moreover, we observed that AIEC indeed had the ability to significantly alter microbiota composition in T5KO but not in WT ASF mice. This disturbance resulted in elevated levels of LPS and flagellin but only modest evidence of intestinal inflammation, suggesting that robust inflammation in T5KO mice requires a complex dysbiotic microbiota.

## Methods

### Ethical statement

All the animal experiments performed in this manuscript were under institutionally-approved protocols: Georgia State’s Institutional Animal Care and Use Committee, IACUC #A14033). Georgia State’s animal welfare assurance number in accordance with the Public Health Service (PHS) policy for humane care and use of laboratory animals is D16-00527 (A3914-01).

Ethics approval and consent to participate. Not applicable.

### Mice

**Fig 1:**
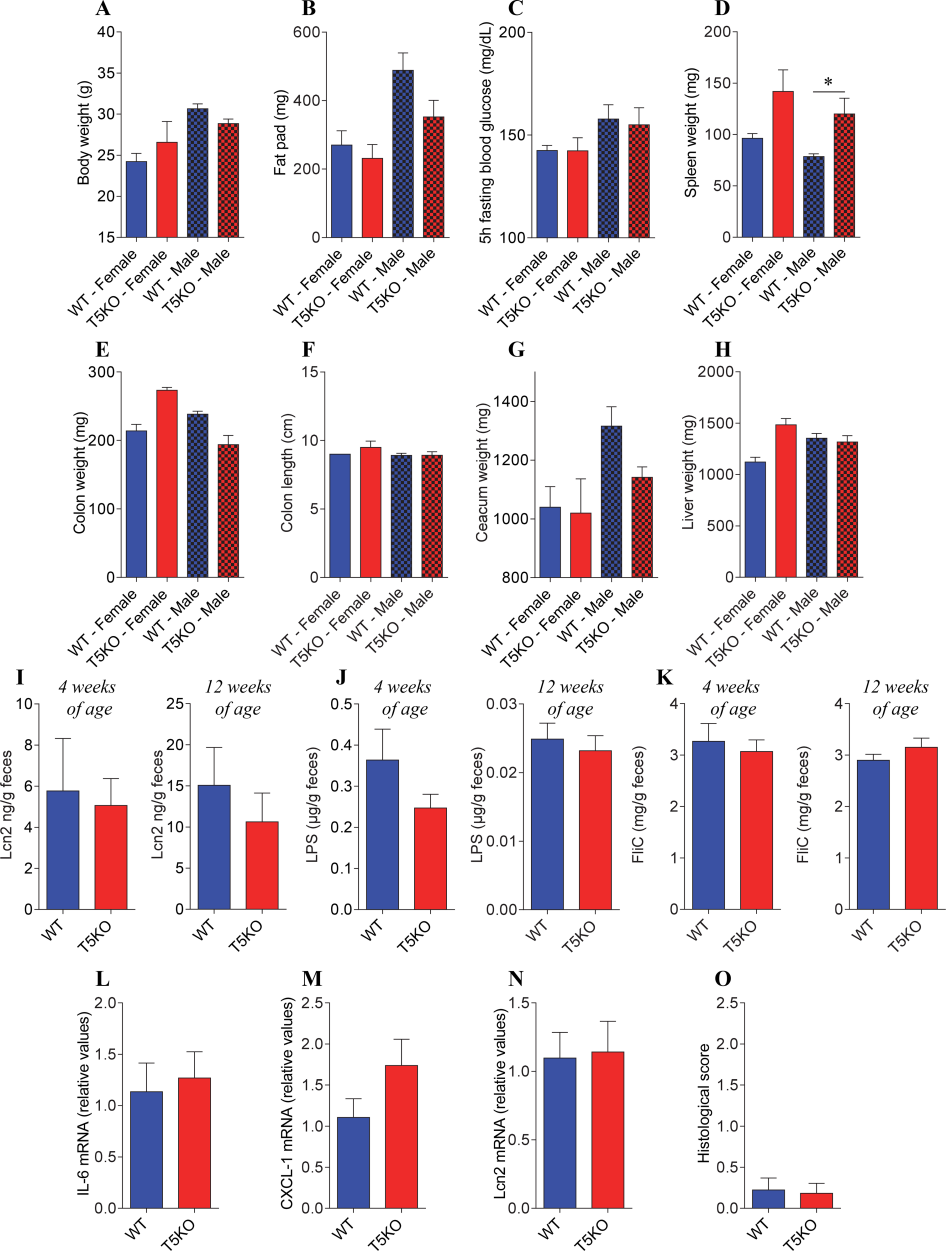
T5KO mice are protected from intestinal inflammation and associated metabolic syndrome when colonized with Altered Schaedler Flora. WT and T5KO C57BL/6 animals, both males and females, were born from mice colonized with the Altered Schaedler Flora and maintained in isolators. At 12 weeks of age, animals were euthanized. **A.** Final body weight. **B.** Fat pad weight. **C.** 5 h fasting blood glucose concentration. **D.** Spleen weight. **E.** Colon weight. **F.** Colon length. **G.** Caecum weight. **H.** Liver weight. **I.** Fecal Lcn2 levels at week 4 and week 12 of age. **J.** Fecal lipopolysaccharide (LPS) levels at week 4 and week 12 of age. **K.** Fecal flagellin (FliC) levels at week 4 and week 12 of age. **L-N.** Colonic pro-inflammatory cytokine-encoding genes were quantified by qRT-PCR (**L**, IL-6; **M**, CXCL-1; **N**, Lcn2). **O.** Hematoxylin & eosin staining was performed on colonic sections and used for the determination of histopathological scores. Data are the means +/- S.E.M. (*n* = 3–6). Significance was determined using *t*-test (* indicates *p*<0.05).

Altered Schaedler Flora (ASF) mice were established by inoculating WT C57BL/6 GF mice with ASF feces purchased from ASF Taconic, Inc. (Hudson, NY), which contained the 8 ASF strains, *via* their drinking water. Briefly, upon receipt and transfer of frozen feces from ASF mice purchased from Taconic, Inc. to our designated isolator, fecal pellets were resuspended in water and placed on animals’ fur and in their drinking water for 7 days, at which point clean water was provided. This approach, despite likely resulting in some oxygen exposure to ASF members, which are known to be stringent anaerobes, was successful in transferring all 8 ASF members to GSU animals (**S1 and S2 Figs**). Thereafter, such “ASF mice” were bred and maintained in a Park Bioservices isolator at Georgia State University, Atlanta, Georgia, USA. All the mice used in this manuscript, except for S1 Fig, were from subsequent generations in order to only use animals that acquired an ASF community at birth. Mice were separated by genotype and fed autoclaved rodent chow # 5021 (LabDiet). Experiments used both male or female mice with comparisons made within gender, as indicated. The number of animals used in each experiment is as follows: Fig 1 and S4 Fig: 3–6; Fig 2 and S6 Fig: 7–9; Fig 3 and S7 Fig: 11–13. Fig 4 and S8 Fig: 7–13; Fig 5 and S9 Fig: 4–13, S1 and S2 Figs: 3–19. Animals were monitored for any adverse clinical signs every day, and we never observed adverse effects before euthanasia.

**Fig 2:**
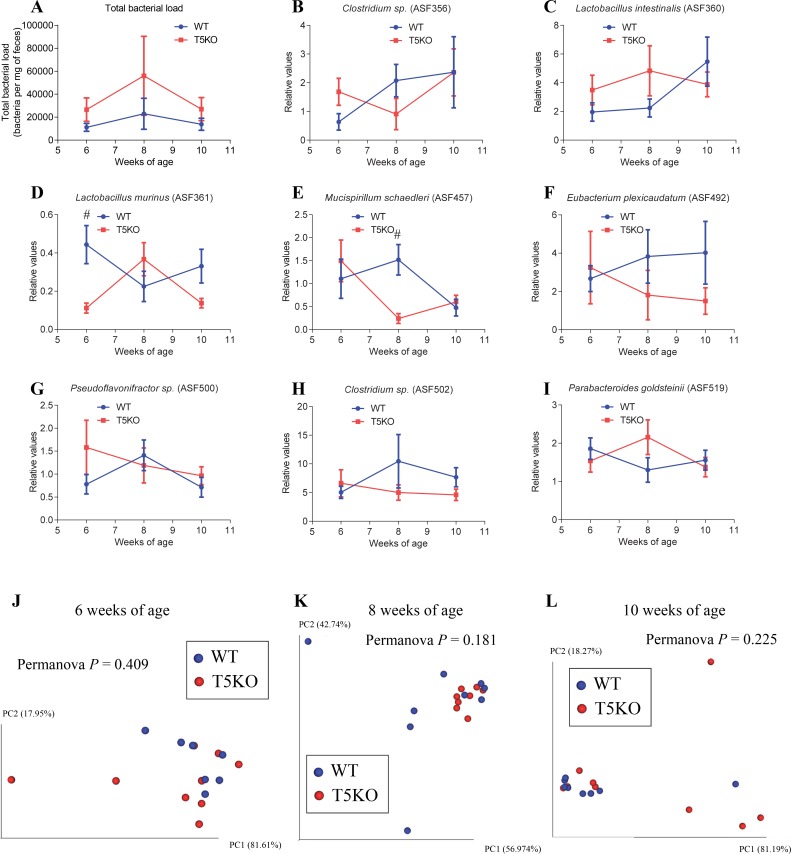
WT and T5KO mice exhibit similar ASF community structure. WT and T5KO mice, both males and females, were born from ASF-colonized mice and maintained in isolators. Feces were collected at weeks 6, 8 and 10 of age. **A.** Total bacterial load. **B.** *Clostridium sp*. (ASF 356) relative values. **C.** *Lactobacillus intestinalis* (ASF 360) relative values. **D.** *Lactobacillus murinus* (ASF 361) relative values. **E.** *Mucispirillum shaedleri* (ASF 457) relative values. **F.** *Eubacterium plexicaudatum* (ASF 492) relative values. **G.** *Pseudoflavonifractor sp*. (ASF 500) relative values. **H.** *Clostridium sp*. (ASF 502) relative values. **I.** *Parabacteroides goldsteinii* (ASF 519) relative values. **J-L** Principal coordinates analysis of the microbiota composition using beta diversity analysis of the Euclidean distance at 6 (**J**), 8 (**K**) and 10 (**L**) weeks of age. *n* = 7–9. Significance was determined using two-way group ANOVA corrected for multiple comparisons with a Bonferroni test (# indicates statistical significance), or Permanova for clustering analysis.

**Fig 3:**
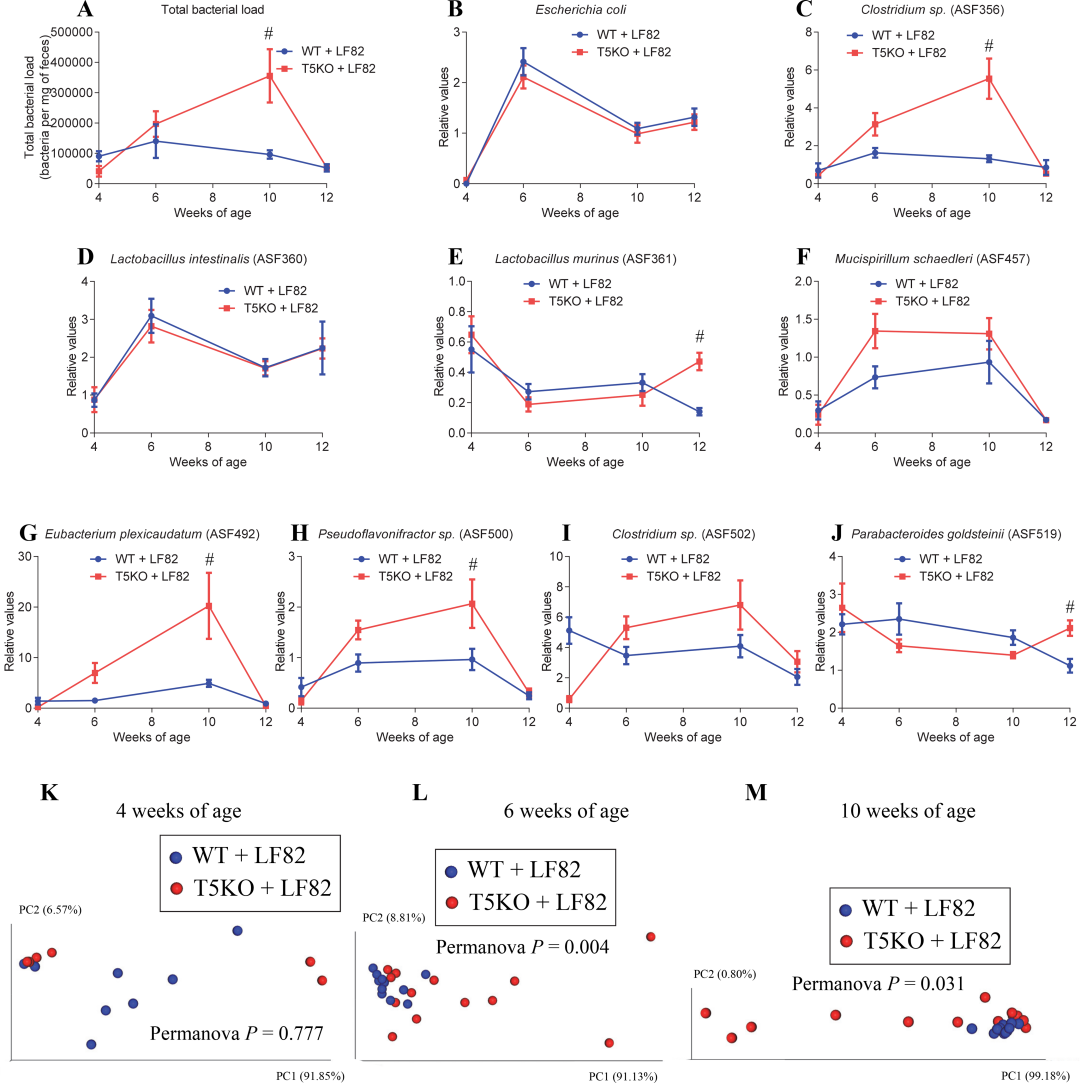
ASF community structure differs between T5KO and WT mice following AIEC colonization. Four-week old offspring of ASF-colonized WT and T5KO mice were removed from the isolator, placed in isolated ventilated cages and inoculated with AIEC reference strain LF82 placed in drinking water for two weeks, followed by return to autoclaved water. Feces were collected at weeks 4, 6, 10 and 12 of age. **A.** Total bacterial load. **B.** *E*. *coli* relative values. **C.** *Clostridium sp*. (ASF 356) relative values. **D.** *Lactobacillus intestinalis* (ASF 360) relative values. **E.** *Lactobacillus murinus* (ASF 361) relative values. **F.** *Mucispirillum shaedleri* (ASF 457) relative values. **G.** *Eubacterium plexicaudatum* (ASF 492) relative values. **H.** *Pseudoflavonifractor sp*. (ASF 500) relative values. **I.** *Clostridium sp*. (ASF 502) relative values. **J.** *Parabacteroides goldsteinii* (ASF 519) relative values. **K-M** Principal coordinates analysis of the microbiota composition using beta diversity analysis of the Euclidean distance at 4 (**J**), 6 (**K**) and 10 (**L**) weeks of age. *n* = 8–13. Significance was determined using two-way group ANOVA corrected for multiple comparisons with a Bonferroni test (# indicates statistical significance), or Permanova for clustering analysis.

**Fig 4:**
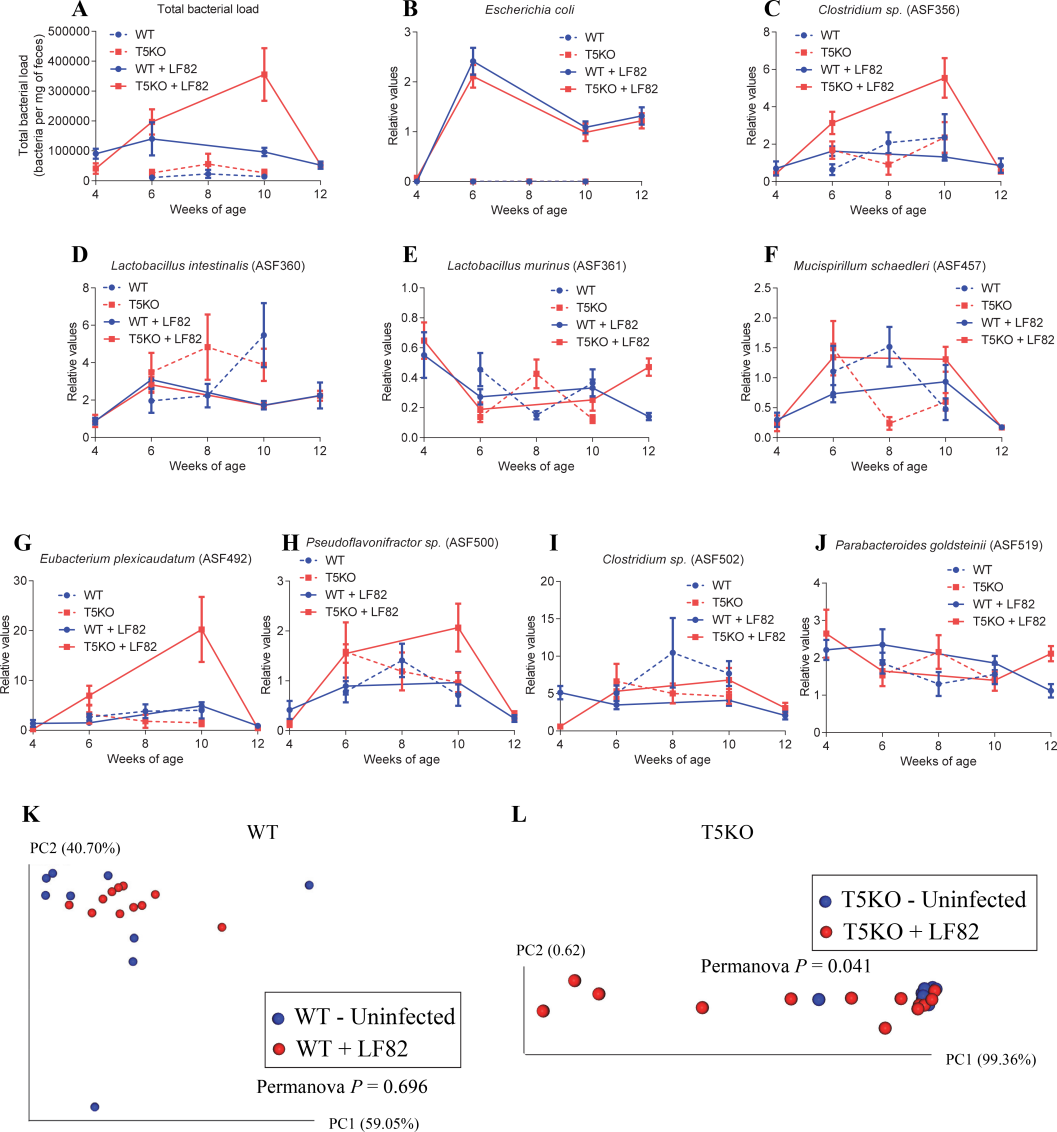
AIEC LF82 colonization alters microbiota composition in T5KO, but not WT, ASF mice. Four-week old offspring of ASF-colonized WT and T5KO mice were remove from the isolator, placed in isolated ventilated cages and inoculated with AIEC reference strain LF82 placed in drinking water for two weeks, followed by return to autoclaved water. Feces were collected at weeks 4, 6, 8, 10 and 12 of age. **A.** Total bacterial load. **B.** *E*. *coli* relative values. **C.** *Clostridium sp*. (ASF 356) relative values. **D.** *Lactobacillus intestinalis* (ASF 360) relative values. **E.** *Lactobacillus murinus* (ASF 361) relative values. **F.** *Mucispirillum shaedleri* (ASF 457) relative values. **G.** *Eubacterium plexicaudatum* (ASF 492) relative values. **H.** *Pseudoflavonifractor sp*. (ASF 500) relative values. **I.** *Clostridium sp*. (ASF 502) relative values. **J.** *Parabacteroides goldsteinii* (ASF 519) relative values. **K-L** Principal coordinates analysis of the microbiota composition using beta diversity analysis of the Euclidean distance in WT ASF (**K**) and T5KO ASF (**L**) animals infected or not with AIEC reference strain LF82. *n* = 8–13. Significance was determined using Permanova for clustering analysis.

**Fig 5:**
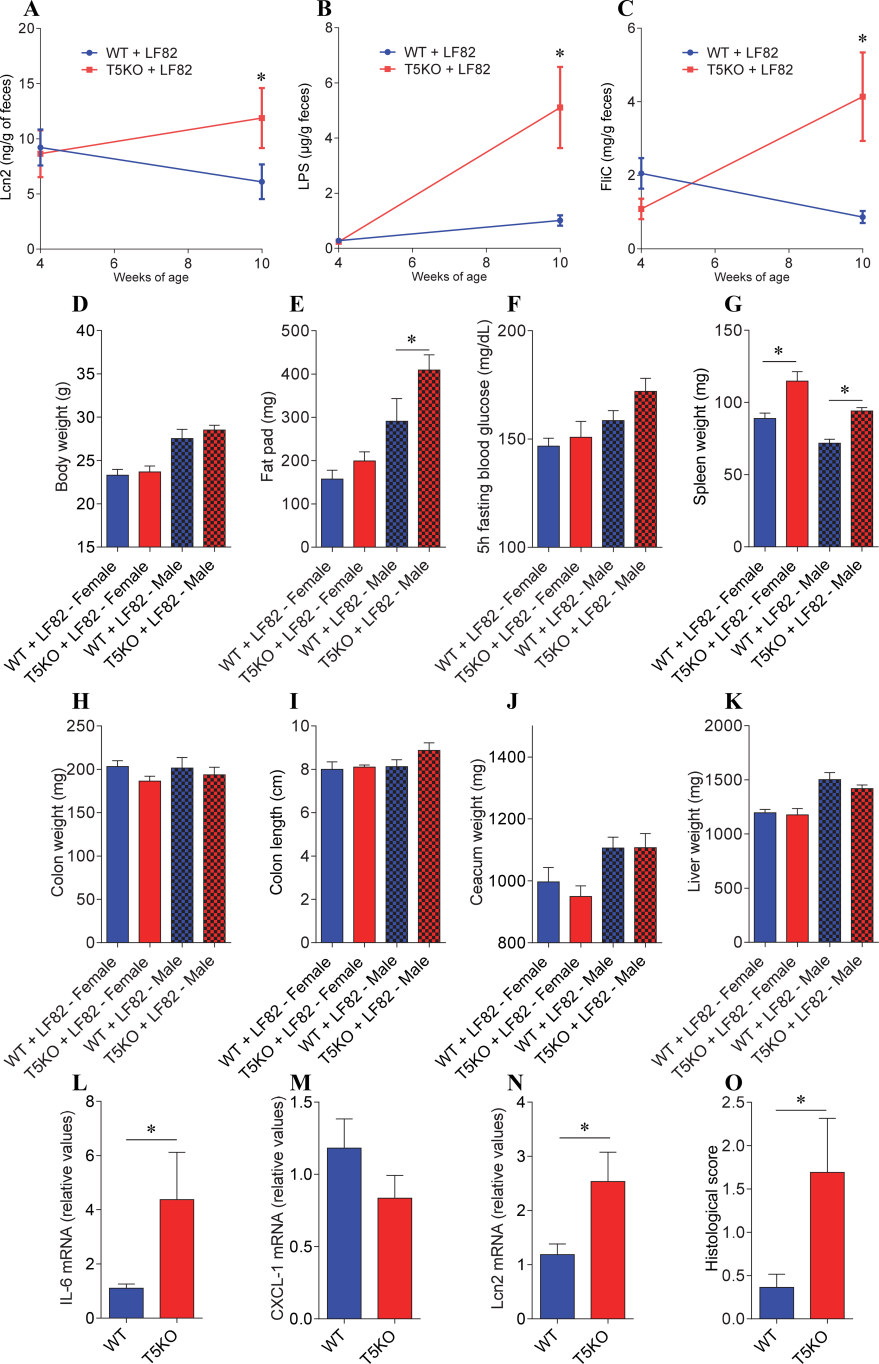
AIEC colonization induces modest increase in low-grade inflammation and adiposity in ASF-T5KO mice. Four-week old offspring of ASF-colonized WT and T5KO mice were removed from the isolator, placed in isolated ventilated cages and inoculated with AIEC reference strain LF82 placed in drinking water for two weeks, followed by return to autoclaved water. At 12 weeks of age, animals were removed from this isolator and euthanized. **A.** Fecal Lcn2 levels at week 4 and week 10 of age. **B.** Fecal lipopolysaccharide (LPS) levels at week 4 and week 10 of age. **C.** Fecal flagellin (FliC) levels at week 4 and week 10 of age. **D.** Final body weight. **E.** Fat pad weight. **F.** 5 h fasting blood glucose concentration. **G.** Spleen weight. **H.** Colon weight. **I.** Colon length. **J.** Caecum weight. **K.** Liver weight. **L-N.** Colonic pro-inflammatory cytokine-encoding genes were quantified by qRT-PCR (**L**, IL-6; **M**, CXCL-1; **N**, Lcn2). **O.** Hematoxylin & eosin staining was performed on colonic sections and used for the determination of histopathological scores. Data are the means +/- S.E.M. (*n* = 3–6). Significance was determined using *t*-test (* indicates *p*<0.05). Data are the means +/- S.E.M. (*n* = 3–6). Data in A, B and C combine both male and female animals. Significance was determined using *t*-test (* indicates *p*<0.05).

### Infection of ASF mice with AIEC strain LF82

AIEC, reference strain LF82 [16], was grown overnight in 200 mL of Luria-Bertani broth at 37°C without agitation. Twenty-four hours post-inoculation, bacterial suspensions, with OD_620nm_ of 2.0, were placed in water bottles of three to four-week-old ASF C57BL/6 mice, which had been placed in isolated ventilated caging system (Isocage, Techniplast, Buguggiate VA, Italy) that prevents exogenous bacterial contamination [24]. Two weeks later, bacterial suspensions were removed and replaced with autoclaved drinking water, using a new sterile bottle. Fresh feces were collected every other week for downstream analysis. Eight-weeks post-inoculation, mice were fasted for 5 h at which time blood was collected by retro-bulbar intra-orbital capillary plexus. Hemolysis-free serum was generated by centrifugation of blood using serum separator tubes (Becton Dickinson, Franklin Lakes, NJ). Mice were then euthanized, and colon length, colon weight, spleen weight and adipose weight measured. Organs were collected for downstream analysis.

### Fasting blood glucose measurement

Mice were placed in a clean cage and fasted for 5 h. Blood glucose concentration was then determined using a Nova Max Plus Glucose Meter (Novacares, Billerica, Massachusetts, USA) and expressed in mg/dL.

### qRT-PCR analysis

Total RNAs were isolated from colonic tissues using TRIzol (Invitrogen, Carlsbad, CA) according to the manufacturer’s instructions. Quantitative RT-PCR were performed using the Qiagen kit QuantiFastH SYBRH Green RT-PCR in a CFX96 apparatus (Bio-Rad, Hercules, CA) with specific mouse oligonucleotides. The sense and antisense oligonucleotides used are presented in **Table 1**, and results were normalized to the housekeeping gene 36B4.

**Table 1:** Primers used in the study [27]. Flagellated bacteria were determined based on [30]. N.A., not applicable.

	ASF Number	Forward (5’ > 3’)	Reverse (5’ > 3’))	Flagellated
EUB	N.A.	515F GTGCCAGCMGCCGCGGTAA	806R GGACTACHVGGGTWTCTAAT	N.A.
*Clostridium sp*.	356	AAAATAATTAGGAGCTTGCTTTTAA	TTAGAAGATGCCTCCTAAGAACC	Yes
*Lactobacillus intestinalis*	360	GGTGATGACGCTGGGAAC	AAGCAATAGCCATGCAGC	No
*Lactobacillus murinus*	361	GAACGAAACTTCTTTATCACC	TAGCATAGCCACCTTTTACA	No
*Mucispirillum shaedleri*	457	TCTCTTCGGGGATGATTAAAC	AACTTTTCCTATATAAACATGCAC	Yes
*Eubacterium plexicaudatum*	492	AATTCCTTCGGGGAGGAAGC	TAAAACCATGCGGTTTTAAAAAC	Yes
*Pseudoflavonifractor sp*.	500	ACGGAGGACCCCTGAAGG	AGCGATAAATCTTTGATGTCC	Yes
*Clostridium sp*.	502	GAGCGAAGCACTTTTTTAGAAC	TTACACCACCTCAGTTTTTACC	Yes
*Parabacteroides goldsteinii*	519	GCAGCACGATGTAGCAATACA	TTAACAAATATTTCCATGTGGAAC	No
*Escherichia coli*	N.A.	CATGCCGCGTGTATGAAGAA	CGGGTAACGTCAATGAGCAAA	Yes
*36B4*	N.A.	TCCAGGCTTTGGGCATCA	CTTTATTCAGCTGCACATCACTCAGA	N.A.
*CXCL-1*	N.A.	TTGTGCGAAAAGAAGTGCAG	TACAAACACAGCCTCCCACA	N.A.
*IL-6*	N.A.	ACAAGTCGGAGGCTTAATTACACAT	TTGCCATTGCACAACTCTTTTC	N.A.
*Lcn2*	N.A.	AAGGCAGCTTTACGATGTACAGC	CTTGCACATTGTAGCTGTGTACC	N.A.

### Hematoxylin & eosin staining of colonic tissue and histopathologic analysis

Following euthanasia, colons were divided in two equivalents pieces and the distal part were fixed in 10% buffered formalin for 24 hours at room temperature and then embedded in paraffin in a non-oriented manner. Tissues were sectioned at 5-mm thickness and stained with hematoxylin & eosin (H&E) using standard protocols. H&E stained slides were assigned four scores based on the degree of epithelial damage and inflammatory infiltrate in the mucosa, submucosa and muscularis/serosa, as previously described [25]. A slight modification was made to this scoring system, as we previously reported [26]: each of the four scores was multiplied by 1 if the change was focal, 2 if it was patchy and 3 if it was diffuse. The 4 individual scores per colon were added, resulting in a total scoring range of 0–36 per mouse. Representative images were selected from 1–2 animals per cage, and presented in **S5 and S10 Figs**.

### Quantification of fecal Lcn-2 by ELISA

For quantification of fecal Lcn-2 by ELISA, frozen fecal samples were reconstituted in PBS containing 0.1% Tween 20 to a final concentration of 100 mg/mL and vortexed for 20 min to get a homogenous fecal suspension [26]. These samples were then centrifuged for 10 min at 14,000 g and 4°C. Clear supernatants were collected and stored at −20°C until analysis. Lcn-2 levels were estimated in the supernatants using Duoset murine Lcn-2 ELISA kit (R&D Systems, Minneapolis, Minnesota, USA) using the colorimetric peroxidase substrate tetramethylbenzidine, and optical density (OD) was read at 450 nm (Versamax microplate reader).

### Fecal flagellin and lipopolysaccharide load quantification

Levels of fecal bioactive flagellin and lipopolysaccharide were determined as previously described [14] using human embryonic kidney (HEK)-Blue-mTLR5 and HEK-BluemTLR4 cells, respectively (Invivogen, San Diego, California, USA) [14]. Fecal material was re-suspended in PBS to a final concentration of 100 mg/mL and homogenized for 10 s using a Mini-Beadbeater-24 without the addition of beads (Biospec, 2400 oscillations/min) to avoid bacteria disruption. Samples were then centrifuged at 8,000 g for 2 min, and supernatants were serially diluted and applied to mammalian cells. Purified *E*. *coli* flagellin and lipopolysaccharide (Sigma, St Louis, Missouri, USA) were used for standard curve determination using HEK-Blue-mTLR5 and HEK-Blue-mTLR4 cells, respectively. After 24 h of stimulation, cell culture supernatants were applied to QUANTI-Blue medium (Invivogen, San Diego, California, USA) and measured alkaline phosphatase activity at 620 nm after 30 min.

### Bacterial quantification by q-PCR

For quantification of total fecal bacterial load and ASF composition analysis, total bacterial DNA was isolated from feces using QIAamp DNA Stool Mini Kit (Qiagen) that utilizes proteinase K for efficient bacterial lysis. DNA was then subjected to quantitative PCR using QuantiFast SYBR Green PCR kit (Biorad) with universal 16S rRNA primers or with specific primers to each member of the ASF defined microbiota (**Table 1**) [27]. Results are expressed as bacteria number per mg of stool, using the standard curve represented **S3 Fig**, or as relative values. A standard curve was generated as follows: bacterial DNA was extracted using QIAamp DNA Stool Mini Kit (Qiagen) from a serially diluted (1:10) overnight *Escherichia coli* culture from which we determined the exact bacterial concentration by plating cultures on LB agar plate. Five μL of DNA was then subjected to quantitative PCR using QuantiFast SYBR Green PCR kit (Biorad) with universal 16S rRNA primers (**Table 1**). Each qPCR reaction was performed in triplicate and average cycle threshold values vs bacterial concentration were plotted (**S3 Fig**) in order to determine the equation to use for bacterial density determination (Y = 1E+10e^-0.737X^, where Y = bacterial density and X = Cycle threshold). **S3 Fig** represents only the 5 dilutions used for non-linear regression analysis. A dilution of 1:20 was used for all experimental samples in order for the cycle threshold (Ct) values to fit within the standard curve (10.13 < Ct < 22.44). Importantly, due to differences in 16S rRNA gene copy numbers between different species of bacteria, calculated values of bacterial density are approximations as the standard curve was generated using *E*. *coli*. Hence, for ASF community members, relative values were determined as follow: first, 16S (515F-806R) Ct values were used to standardize other values by calculating a ΔCT in order to avoid that an increase bacterial load drive increase in all ASF members relative values, an approach designed to examine proportion of each ASF member within the community rather than their absolute abundance. Subsequently, for each bacterium, one animal (TLR5KO + LF82, week 4 = before LF82 inoculation for all figures except panels 3B, 4B, S6B and S7B where TLR5KO + LF82, week 6 = after LF82 inoculation was used) was determined as 1 and values in other animals were expressed as relative values using the ΔCT approach. For example, if Ct values for *Clostridium sp*. (ASF 356) for 4 animals are A = 22, B = 18, C = 25 and D = 23, and if Ct values for 515F/806R primers are A = 12, B = 11, C = 14 and D = 13, ΔCT values are A = 10, B = 7, C = 11 and D = 10. Accordingly, if animal A is determined as 1, ΔCT values are A = 0, B = 3, C = -1 and D = 0 and relative values are A = 1, B = 8, C = 0.5 and D = 1. Principal coordinate analysis was performed using Euclidean distance with the 8 ASF members (**Figs 2 and 3**) or with the 8 ASF members + AIEC LF82 strain (**Fig 4**). In S1 and S2 Figs, negative controls were generated by applying the DNA extraction protocol to water samples.

### Statistical analysis

Significance were determined using *t*-test or two-way repeated measures ANOVA with Bonferroni multiple comparisons test (GraphPad Prism software, version 6.01). Differences were noted as significant *p≤0.05 for *t*-test and #p≤0.05 for two-way ANOVA. For clustering analyses on principal coordinate plots, categories were compared and statistical significance of clustering were determined via Permanova. S4, S5, S6, S7 and S8 Figs are duplicate of Figs 1, 2, 3, 4 and 5, respectively, but using dot plot representations.

## Results

### T5KO mice do not develop intestinal inflammation nor metabolic syndrome when maintained in Altered Schaedler Flora (ASF)-restricted conditions.

The microbiota-dependency of previously characterized TLR5 phenotypes could indicate their reliance upon an aspect of immune system development or metabolism that simply does not occur in germfree mice or, as we hypothesize, reflect that T5KO mice fail to efficiently control motile pathobionts, which then instigate broader disruptions of the complex microbiota. To investigate these possibilities, germfree WT and T5KO C57BL/6 mice were transferred to a Park Bioservices isolator in our gnotobiotic facility, as previously described [7], and then colonized with the 8 strains of bacteria that comprise the Altered Schaedler Flora (ASF), considered to have minimal pathogenic characteristics but yet sufficient for relatively normal metabolism and immune development. We first validated that all ASF members had indeed colonized our colony by performing q-PCR using specific primers that were recently developed for each ASF member [27]. As presented in **S1 and S2 Figs** (S1 Fig represent cycle threshold and S2 Fig represent relative values normalized with feces weight), we observed that all 8 ASF members had efficiently colonized our WT germfree mice to levels similar to what was observed at Taconic, Inc. Moreover, quantification of total bacterial loads indicated that ASF animals harbor a lower bacterial load compared to conventional animals, suggesting that a complex microbiota might be required to achieve the bacterial load normally found in the intestinal tract (**S2 Fig**), possibly reflecting the presence of unoccupied niches in ASF-colonized mice [20, 21].

We next compared offspring of WT and T5KO ASF mice for potential differences in phenotype and microbiota composition (**Figs 1 and 2 and S4, S5 and S6 Figs**). In contrast to the case of conventional conditions [5, 10, 13], T5KO mice bred and maintained under ASF-restricted conditions did not exhibit evidence of low-grade intestinal inflammation compared to WT mice, as assessed by levels of fecal Lcn2, which is a sensitive and dynamic marker of gut inflammation [26]. Interestingly, fecal Lcn2 levels in ASF animals were intermediate between germfree animals (close to 0 ng/g of feces) and conventional animals (around 30 ng/g of feces,), suggesting that the significant but lower than normal bacterial loads in these mice resulted in an intestinal innate immune system that was not fully mature but yet more active than in germfree animals (**Fig 1I and S4 Fig**). Colon or caecum morphology, which can also reflect low-grade inflammation, was not altered in T5KO ASF mice relative to WT ASF animals (**Fig 1E–1G and S4 Fig**), while livers were slightly enlarged in T5KO female mice compared to WT females. (**Fig 1H and S4 Fig**). Colonic pro-inflammatory cytokines expression and histological scores were similar between WT ASF and T5KO ASF (**Fig 1L–1O, S4 and S5 Figs**). T5KO ASF mice were not completely absent from evidence of systemic inflammation in that their spleens were moderately but significantly enlarged compared to WT ASF mice. (**Fig 1D and S4 Fig**). Nonetheless, consistent with their lack of evidence of low-grade intestinal inflammation, T5KO ASF mice exhibited no evidence of metabolic syndrome, as assessed by body weight, fat mass, or fasting glucose levels at 12 weeks of age (**Fig 1 and S4 Fig**), at which time these parameters were consistently altered in T5KO compared to WT animals under conventional conditions [5, 13]. We next measured levels of bioactive fecal flagellin and LPS, which we have previously reported are a relative reflection of a microbiota’s pro-inflammatory potential and are both elevated in mice lacking TLR5 [13]. We utilized an assay that measures the ability of un-lysed fecal supernatant to activate TLR5 and TLR4 reporter cells. Such levels of bioactive flagellin and LPS can be modulated either at the level of gene expression or as a consequence of microbiota community structure shift, since different bacteria make different types and levels of flagellin and LPS, which can differentially activate TLR5 and TLR4 receptors [28, 29].

Of note, of the 8 ASF community members, *Clostridium sp*. (ASF 356), *Mucispirillum shaedleri* (ASF 457), *Eubacterium plexicaudatum* (ASF 492), *Eubacterium plexicaudatum* (ASF 500) and *Clostridium sp*. (ASF 502) have the capacity to make flagellin [30]. As presented in **Fig 1J and 1K**, levels of LPS and flagellin were similar between T5KO and WT animals at both post-weaning (4 weeks-old) and 12 weeks-old. Altogether, such data indicates that, similarly to what was observed under germfree conditions, T5KO mice raised and maintained in ASF condition lack low-grade intestinal inflammation and metabolic syndrome phenotypes previously observed under conventional conditions.

### WT and T5KO mice exhibit similar ASF community structure

We next investigated microbiota composition in WT and T5KO animals harboring the ASF community. We observed that T5KO mice displayed a modest, albeit not significant, increase in total bacterial loads relative to ASF WT mice, analogous to previous observations in conventional mice that suggested that optimal management of microbial community requires TLR5 [5, 31] (**Fig 2A and S6A Fig**). Yet, little difference was observed in the overall community structure. Specifically, while a few differences for some ASF members were observed, such as a decrease in *Lactobacillus murinus* (ASF 361) and *Mucispirillum shaedleri* (ASF 457) in T5KO animals compared to WT animals at 6 and 8 weeks of age, respectively (**Fig 2D and 2E**), principal coordinate representation of the 8 ASF members revealed no major clustering of T5KO mice compared to WT controls, suggesting that the overall ASF community is unaltered in T5KO mice (**Fig 2J–2L**, Permanova values: 0.409, 0.181, 0.225 at 6, 8 and 10 weeks of age, respectively). Moreover, we did not observe any sex effect on the ASF community structure (data not shown). Such ability of T5KO mice to manage ASF microbiota similar to WT mice may explain their lack of low-grade intestinal inflammation and metabolic syndrome.

### Adherent and Invasive Escherichia coli alters the overall microbiota community structure in T5KO ASF but not WT ASF mice

Administration of the Crohn’s disease-associated *E*. *coli* strain AIEC LF82 [16, 17] to T5KO mice during the post-weaning period results in a disturbance of the overall microbiota composition that persists long after the added bacterium was cleared [10, 14]. Hence, we next investigated if AIEC LF82 inoculation might alter ASF community in T5KO relative to WT mice. AIEC LF82 was grown overnight in Luria-Bertani broth, and bacterial suspensions were placed in drinking water of four weeks-old ASF mice. Mice were maintained in an isolated ventilated caging system (Isocages) in order to prevent exposure to environmental bacteria, as previously described [24, 32, 33]. Total bacterial loads were significantly increased in T5KO mice following AIEC administration while they remained stable in WT mice (**Fig 3A and S7A Fig**), with levels of the AIEC flagellated pathobiont not being significantly altered between WT and T5KO mice at any of the time points assayed (2, 6, and 8-weeks post-inoculation, **Fig 3B**). Such increase in total bacterial loads was mainly driven by significant elevations in levels of *Clostridium sp*. (ASF 356), *Eubacterium plexicaudatum* (ASF 492) and *Pseudoflavonifractor sp*. (ASF 500) in T5KO compared to WT animals (**Fig 3C, 3G and 3H**). Other perturbations of the ASF community were observed in T5KO mice compared to WT mice following AIEC inoculation, such as an increase in *Lactobacillus murinus* (ASF 361) and *Parabacteroides goldsteinii* (ASF 519) (**Fig 3E and 3J**). Examining the global community composition using principal coordinate analysis revealed an alteration of the ASF community structure in T5KO mice compare to WT controls at 2 and 6 weeks post-AIEC inoculation (6 and 10 weeks of age, respectively) (**Fig 3L and 3M**). While we previously reported that AIEC LF82 bacteria are not able to stably colonize WT or T5KO mice [10], we observed here that they can stably colonize ASF-colonized animals, in accord with previous observations that such low complexity microbial community is not sufficient to confer full colonization resistance [20].

Combined analysis of the 4 groups of mice (WT, WT + AIEC LF82, T5KO and T5KO + AIEC LF82) revealed that T5KO carrying AIEC exhibited major differences in the ASF composition compared to the 3 other groups, characterized by a strong increase in total bacterial load, and increases in *Clostridium sp*. (ASF 356) and *Eubacterium plexicaudatum* (ASF 492) (**Fig 4A–4J and S8 Fig**). Concomitantly, comparison of T5KO mice that had or had not been inoculated with AIEC revealed a significant difference in microbiota composition between these groups (**Fig 4L,** Permanova value: 0.041), while no such difference was observed between WT mice that had or had not received AIEC (**Fig 4K**).

Altogether, these results indicate that, analogous to the case of conventional mice, post-weaning administration of AIEC LF82 to ASF induces lasting changes in microbial community structure in T5KO but not WT mice. It should be noted that the initial comparison of WT versus TLR5KO animals (**Figs 1 and 2**) was performed in a traditional gnotobiotic isolator while the experiment comparing WT and TLR5KO animals after the introduction of AIEC LF82 bacteria (**Fig 3**) was performed using isolated and ventilated caging system (details in Methods). The combined representation of both experiments in **Fig 4** reveal that, in two independent experiments, performed in two different types of isolator units, WT animals have similar ASF composition, further highlighting the stability of this defined community.

### AIEC administration induces modest level of low-grade gut inflammation in T5KO mice relative to similarly treated WT mice

The AIEC-induced disturbance of T5KO microbiota composition previously observed in mice with a complex microbiota correlates with marked elevations in levels of LPS and flagellin and, moreover clear histopathologic evidence of intestinal inflammation [10, 14]. Hence, we next investigated if analogous events occurred in mice with the relatively simple ASF microbiota used here. Prior to AIEC administration (4 weeks of age), WT and TLR5 ASF mice exhibited similar levels of bioactive LPS and flagellin, whereas T5KO mice exhibited higher levels of both microbial products 6 weeks following AIEC administration (**Fig 5B and 5C and S9 Fig**). Analogously, fecal Lcn2 levels did not differ between WT and T5KO ASF mice prior to AIEC administration, but were modestly elevated by 10 weeks of age, suggesting that T5KO mice may have developed low-grade inflammation relative to similarly-treated WT mice (**Fig 5A**). Such T5KO mice did not exhibit alterations in colon weight and length, but did exhibit significant increase in the expression of genes encoding pro-inflammatory cytokines, which correlated with a modest but significant increase in colonic histopathological scores (**Fig 5L–5O, S9 and S10 Figs**). Moreover, following LF82 infection, T5KO ASF mice developed mild indications of metabolic syndrome, namely a trend toward increased adiposity 6 weeks following presence of AIEC (reached statistical significance for male but not female mice, **Fig 5E**). Further analysis revealed that *Lactobacillus murinus* (ASF 361) and *Parabacteroides goldsteinii* (ASF 519) abundances correlated with final body and fat pad weights, and that *Clostridium sp*. (ASF 356) *Parabacteroides goldsteinii* (ASF 519) and *Lactobacillus murinus* (ASF 361) abundances correlated with makers of intestinal inflammation (spleen weight, colon weight and fecal lipocalin-2, respectively, **S11 Fig**). While such correlations may link some ASF community members to host physiology, further studies would be needed to assess if such alterations in relative abundances are playing a role in the phenotypes observed or if they are largely a consequence of either AIEC LF82 inoculation and/or development of low-grade inflammation. To conclude, in contrast to the case of a complex microbiota, the disturbance of the simple ASF microbiota induced by AIEC LF82 had only a modest impact upon the host.

## Discussion

Ability to rederive and maintain mice in a germfree state provides a powerful tool to unequivocally determine the extent to which phenotypes observed in specific mouse strains, engineered or naturally-occurring, are dependent upon the presence of a microbiota. However, for phenotypes that prove to be microbiota-dependent, germfree mice offer little options to mechanistically discern how/why the presence of a microbiota in that strain results in the observed phenotype. On the other hand, the great complexity of non-engineered intestinal microbiomes in which there is a high degree of heterogeneity within and amongst mouse strains, combined with the bi-directional impact that inflammation and altered microbiota composition have on each other, makes mechanistic elucidation of the host-microbiota relationship in inflammatory diseases a great challenge. Hence, one approach that has been used to bridge this gap are mice with a limited defined microbiota such as the Altered Schaedler Flora (ASF), which we used herein. We observed that despite 5 of 8 ASF species being flagellated, loss of TLR5 did not have a significant impact on the ASF community structure. Addition of a single pathobiont *E*. *coli* strain induced a moderate disturbance of this community that correlated with a modest level of gut inflammation in T5KO mice, whereas administration of this pathobiont to T5KO mice with a complex microbiota caused a more dramatic change in microbiota composition that associated with robust intestinal inflammation [10, 14]. In a similar vein, we recently reported that a non-genetic perturbant of the microbiota, namely emulsifiers, also did not alter the microbiota nor induce low-grade inflammation in ASF mice [7, 33]. Importantly, neither our past or present findings dispute the commonly held assumption that excess caloric consumption and unbalanced energy intake vs. expenditure is a predominant factor that drive metabolic syndrome, but rather, we suggest that such hyperphagia may be driven, at least in part, by alterations of the gut microbiota promoting low-grade intestinal inflammation.

Increased levels of fecal LPS and flagellin were observed following AIEC inoculation of T5KO mice, while AIEC load was observed to be similar between WT and T5KO animals. One possible explanation underlying this observation is that the administered AIEC overexpress these pro-inflammatory molecules in T5KO mice. However, we speculate that, in fact, enrichment of flagellated ASF members *Clostridium sp*. (ASF 356) and *Eubacterium plexicaudatum* (ASF 492) following AIEC LF82 colonization might be responsible for these increased levels of fecal LPS and flagellin. Considering these results and ideas together suggests that the spontaneous chronic phenotypes observed in T5KO mice may not reflect outgrowth of a single species but rather may result from a more general inability to maintain a stable complex microbiome in the context of a pathobiont infection. Hence, such findings further highlight the importance played by the intestinal microbiota and pathobiont in the development of intestinal inflammation [34, 35]. While our data support the notion that pathobiont infection detrimentally alters the intestinal microbiota, potentially promoting intestinal inflammation, diet can play a central role in microbiota stability and pathobiont expansion, with for example the observation that saturated (milk-derived) fat is sufficient to induce a bloom in *Bilophila wadsworthia*, that drives intestinal inflammation in IL10-deficient mice [36].

It remains unclear whether the low-grade intestinal inflammation observed following AIEC administration to ASF mice is a consequence of AIEC inoculation, its induction of changes in the ASF microbiota alteration (flagellated ASF members are increased following AIEC LF82 infection), or both. While the observation that T5KO animals mono-associated with AIEC developed some degree of intestinal inflammation [10] suggests that AIEC is playing a direct role in this phenotype, we also previously reported that T5KO mice administered AIEC were only transiently colonized by AIEC but yet developed intestinal inflammation that persisted well after AIEC disappearance from the intestinal tract, suggesting that alterations in microbiota composition contribute to intestinal inflammation [14] in these mice. Hence, deciphering if flagellated ASF members are participating in intestinal inflammation or if they simply benefit from inflammation induced by AIEC will require further investigations. One possible future approach could be to develop a means of reversible AIEC inoculation, analogous to previous method used for non-pathogenic *E*. *coli* [37], that will allow to investigate the impact of altered microbiota versus AIEC *per se*.

It is not currently clear why a complex microbiota is required for manifestation of phenotypes associated with TLR5 deficiency, but several possible reasons can be envisaged. One relatively basic reason might be that the total bacterial density is more than 2-fold lower in ASF mice than in conventional mice. Hence, it is difficult to exclude the possibility that such greater bacterial density in the gut plays a role in maximizing the microbiota’s pro-inflammatory potential. Another possibility that should be considered is that the gut physiology of ASF mice remains abnormal. In particular, we observed that colons of ASF mice (WT and T5KO) were somewhat fragile and had thin poorly-developed mucus (data not shown). While these features might tend to promote inflammation, particularly upon challenge by a pathobiont such as AIEC, it is possible that it permits microbiota encroachment in a way that masks relative differences in WT and T5KO mice maintained in ASF conditions (such features of ASF colon tissue preclude us from assessing microbiota encroachment by non-dehydrating fixation, as performed in some of our recent publications [7]). Such caveats notwithstanding, we speculate that, more likely, a more complex microbiota is difficult to corral, thus requiring greater immune pressure (innate and perhaps adaptive) to result in a stable community, and that host mediators of this response (i.e. cytokines) interfere with metabolic signaling thus promoting metabolic syndrome and, in select circumstances, resulting in chronic immune cell infiltration that characterizes colitis.

## Conclusion

To conclude, we herein observed that in mice with a limited-complexity pathobiont-free microbiota, loss of the flagellin receptor TLR5 does not significantly impact microbiota composition nor its ability to promote inflammation. Addition of AIEC to this ecosystem perturbs microbiota composition, increases levels of bioactive lipopolysaccharide and flagellin, but only modestly promotes some parameters of intestinal inflammation and adiposity, suggesting that the phenotypes previously associated with loss of this innate immune receptor require disruption of a complex microbiota. Future work with other defined microbiotas, perhaps of medium complexity, will be needed to further define and advance this concept.

## Supporting information

S1 Fig Georgia State University ASF mice are efficiently colonized by all 8 ASF members. Feces were collected from conventional WT C57BL/6 and from WT C57BL/6 animals kept under ASF conditions at Taconic facility or at Georgia State University. **A.** Total bacterial load (515F-806R) cycle threshold values. **B.** *Clostridium sp*. (ASF 356) cycle threshold values. **C.** *Lactobacillus intestinalis* (ASF 360) cycle threshold values. **D.** *Lactobacillus murinus* (ASF 361) cycle threshold values. **E.** *Mucispirillum shaedleri* (ASF 457) cycle threshold values. **F.** *Eubacterium plexicaudatum* (ASF 492) cycle threshold values. **G.** *Pseudoflavonifractor sp*. (ASF 500) cycle threshold values. **H.** *Clostridium sp*. (ASF 502) cycle threshold values. **I.** *Parabacteroides goldsteinii* (ASF 519) cycle threshold values. *n* = 3–19. Significance was determined using *t*-test (* indicates *p*<0.05).
(PDF)

S2 Fig Georgia State University ASF mice are efficiently colonized by all 8 ASF members. Feces were collected from conventional WT C57BL/6 and from WT C57BL/6 animals kept under ASF conditions at Taconic facility or at Georgia State University. **A.** Total bacterial load (515F-806R) express in bacteria per g of feces. **B.** *Clostridium sp*. (ASF 356) relative values, normalized with feces weight. **C.** *Lactobacillus intestinalis* (ASF 360) relative values, normalized with feces weight. **D.** *Lactobacillus murinus* (ASF 361) relative values, normalized with feces weight. **E.** *Mucispirillum shaedleri* (ASF 457) relative values, normalized with feces weight. **F.** *Eubacterium plexicaudatum* (ASF 492) relative values, normalized with feces weight. **G.** *Pseudoflavonifractor sp*. (ASF 500) relative values, normalized with feces weight. **H.** *Clostridium sp*. (ASF 502) relative values, normalized with feces weight. **I.** *Parabacteroides goldsteinii* (ASF 519) relative values, normalized with feces weight. *n* = 3–19. Significance was determined using *t*-test (* indicates *p*<0.05).
(PDF)

S3 Fig Standard curve used for total bacterial density determination. Bacterial DNA was extracted using QIAamp DNA Stool Mini Kit (Qiagen) from a serially diluted (1:10) overnight *Escherichia coli* culture from which we determined the exact bacterial concentration by plating bacterial culture on LB agar plate. Five μL of DNA was then subjected to quantitative PCR using QuantiFast SYBR Green PCR kit (Biorad) with universal 16S rRNA primers. Negative control = DNA extraction protocol was applied on water samples. Cycle threshold values vs bacterial concentration were plotted in order to determine the equation to use for bacterial density determination (Y = 1E+10e^-0.737X^). Non-linear (X is linear, Y is exponential) regression line was draft and R^2^ was determined.
(PDF)

S4 Fig T5KO mice are protected from intestinal inflammation and associated metabolic syndrome when colonized with Altered Schaedler Flora. WT and T5KO C57BL/6 mice, both males and females, were born from mice colonized with the Altered Schaedler Flora and maintained in isolators. At 12 weeks of age, mice were euthanized. **A.** Final body weight. **B.** Fat pad weight. **C.** 5 h fasting blood glucose concentration. **D.** Spleen weight. **E.** Colon weight. **F.** Colon length. **G.** Caecum weight. **H.** Liver weight. **I.** Fecal Lcn2 levels at week 4 and week 12 of age. **J.** Fecal lipopolysaccharide (LPS) levels at week 4 and week 12 of age. **K.** Fecal flagellin (FliC) levels at week 4 and week 12 of age. **L-N.** Colonic pro-inflammatory cytokine-encoding genes were quantified by qRT-PCR (**L**, IL-6; **M**, CXCL-1; **N**, Lcn2). **O.** Hematoxylin & eosin staining was performed on colonic sections and used for the determination of histopathological scores. Each dot represents one animals, bars represent means +/- S.E.M.. The data presented here are the same as Fig 1. (*n* = 3–6). Significance was determined using *t*-test (* indicates *p*<0.05).
(PDF)

S5 Fig T5KO mice are protected from intestinal inflammation and associated metabolic syndrome when colonized with Altered Schaedler Flora. WT and T5KO C57BL/6 mice, both males and females, were born from mice colonized with the Altered Schaedler Flora and maintained in isolators. At 12 weeks of age, mice were euthanized. Hematoxylin & eosin staining was performed on colonic sections, and representative images were selected from 1–2 animals per cage. White boxes indicate individual histological score.
(PDF)

S6 Fig WT and T5KO mice exhibit similar ASF community structures. WT and T5KO mice, both males and females, were born from ASF-colonized mice and maintained in isolators. Feces were collected at weeks 6, 8 and 10 of age. **A.** Total bacterial load. **B.** *Clostridium sp*. (ASF 356) relative values. **C.** *Lactobacillus intestinalis* (ASF 360) relative values. **D.** *Lactobacillus murinus* (ASF 361) relative values. **E.** *Mucispirillum shaedleri* (ASF 457) relative values. **F.** *Eubacterium plexicaudatum* (ASF 492) relative values. **G.** *Pseudoflavonifractor sp*. (ASF 500) relative values. **H.** *Clostridium sp*. (ASF 502) relative values. **I.** *Parabacteroides goldsteinii* (ASF 519) relative values. Each dot represents one animals, bars represent mean. The data presented here are the same as Fig 2. *n* = 7–9. Significance was determined using two-way group ANOVA corrected for multiple comparisons with a Bonferroni test (# indicates statistical significance).
(PDF)

S7 Fig Altered ASF community structures between T5KO and WT mice following AIEC colonization. Four-week old offspring of ASF-colonized WT and T5KO mice were removed from the isolator, placed in isolated ventilated cages and inoculated with AIEC reference strain LF82 placed in drinking water for two weeks, followed by return to autoclaved water. Feces were collected at weeks 4, 6, 10 and 12 of age. **A.** Total bacterial load. **B.** *E*. *coli* relative values. **C.** *Clostridium sp*. (ASF 356) relative values. **D.** *Lactobacillus intestinalis* (ASF 360) relative values. **E.** *Lactobacillus murinus* (ASF 361) relative values. **F.** *Mucispirillum shaedleri* (ASF 457) relative values. **G.** *Eubacterium plexicaudatum* (ASF 492) relative values. **H.** *Pseudoflavonifractor sp*. (ASF 500) relative values. **I.** *Clostridium sp*. (ASF 502) relative values. **J.** *Parabacteroides goldsteinii* (ASF 519) relative values. Each dot represents one animals, bars represent mean. The data presented here are the same as Fig 3. *n* = 8–13. Significance was determined using two-way group ANOVA corrected for multiple comparisons with a Bonferroni test (# indicates statistical significance).
(PDF)

S8 Fig AIEC LF82 colonization alters microbiota composition in T5KO, but not WT, ASF mice. Four-week old offspring of ASF-colonized WT and T5KO mice were remove from the isolator, placed in isolated ventilated cages and inoculated with AIEC reference strain LF82 placed in drinking water for two weeks, followed by return to autoclaved water. Feces were collected at weeks 4, 6, 8, 10 and 12 of age. **A.** Total bacterial load. **B.** *E*. *coli* relative values. **C.** *Clostridium sp*. (ASF 356) relative values. **D.** *Lactobacillus intestinalis* (ASF 360) relative values. **E.** *Lactobacillus murinus* (ASF 361) relative values. **F.** *Mucispirillum shaedleri* (ASF 457) relative values. **G.** *Eubacterium plexicaudatum* (ASF 492) relative values. **H.** *Pseudoflavonifractor sp*. (ASF 500) relative values. **I.** *Clostridium sp*. (ASF 502) relative values. **J.** *Parabacteroides goldsteinii* (ASF 519) relative values. Each dot represents one animals, bars represent mean. The data presented here are the same as Fig 4. *n* = 8–13.
(PDF)

S9 Fig AIEC colonization induces a modest level low-grade inflammation and adiposity in ASF-T5KO mice. Four-week old offspring of ASF-colonized WT and T5KO mice were removed from the isolator, placed in isolated ventilated cages and inoculated with AIEC reference strain LF82 placed in drinking water for two weeks, followed by return to autoclaved water. At 12 weeks of age, animals were removed from this isolator and euthanized. **A.** Fecal Lcn2 levels at week 4 and week 10 of age. **B.** Fecal lipopolysaccharide (LPS) levels at week 4 and week 10 of age. **C.** Fecal flagellin (FliC) levels at week 4 and week 10 of age. **D.** Final body weight. **E.** Fat pad weight. **F.** 5 h fasting blood glucose concentration. **G.** Spleen weight. **H.** Colon weight. **I.** Colon length. **J.** Caecum weight. **K.** Liver weight. **L-N.** Colonic pro-inflammatory cytokine-encoding genes were quantified by qRT-PCR (**L**, IL-6; **M**, CXCL-1; **N**, Lcn2). **O.** Hematoxylin & eosin staining was performed on colonic sections and used for the determination of histopathological scores. Each dot represents one animals, bars represent means +/- S.E.M.. The data presented here are the same as Fig 5. (*n* = 3–6). Data in A, B and C combine both male and female animals. Significance was determined using *t*-test (* indicates *p*<0.05).
(PDF)

S10 Fig AIEC colonization induces a modest level low-grade inflammation and adiposity in ASF-T5KO mice. Four-week old offspring of ASF-colonized WT and T5KO mice were removed from the isolator, placed in isolated ventilated cages and inoculated with AIEC reference strain LF82 placed in drinking water for two weeks, followed by return to autoclaved water. At 12 weeks of age, animals were removed from this isolator and euthanized. Hematoxylin & eosin staining was performed on colonic sections, and representative images were selected from 1–2 animals per cage. White boxes indicate individual histological score.
(PDF)

S11 Fig Correlation analysis between ASF members and parameters of metabolic syndrome and intestinal inflammation. Four-week old offspring of ASF-colonized WT and T5KO mice were removed from the isolator, placed in isolated ventilated cages and inoculated with AIEC reference strain LF82 placed in drinking water for two weeks, followed by return to autoclaved water. **A.** Relative values of *Lactobacillus murinus* (ASF 361) at week 12 and final body weights were plotted in X and Y axis, respectively. **B.** Relative values of *Lactobacillus murinus* (ASF 361) at week 12 and fat pad weights were plotted in X and Y axis, respectively. **C.** Relative values of *Parabacteroides goldsteinii* (ASF 519) at week 12 and fat pad weights were plotted in X and Y axis, respectively. **D.** Relative values of *Clostridium sp*. (ASF 356) at week 12 and spleen weights were plotted in X and Y axis, respectively. **E.** Relative values of *Parabacteroides goldsteinii* (ASF 519) at week 12 and colon weights were plotted in X and Y axis, respectively. **F.** Relative values of *Lactobacillus murinus* (ASF 361) at week 10 and final fecal Lcn2 levels were plotted in X and Y axis, respectively. Linear regression lines were drafted and R^2^ were determined. (*n* = 11).
(PDF)

## Abbreviations

AIEC: adherent-invasive *Escherichia coli*
ASF: Altered Schaedler Flora
FliC: flagellin
IBD: inflammatory bowel disease
LPS: lipopolysaccharide
TLR5: Toll like receptor 5
