# Supplementary figures and images for: Mice harboring pathobiont-free microbiota do not develop intestinal inflammation that normally results from an innate immune deficiency
Source: PLoS One. 2018 Apr 4;13(4):e0195310. doi: 10.1371/journal.pone.0195310 (PMC5884553; doi:10.1371/journal.pone.0195310)

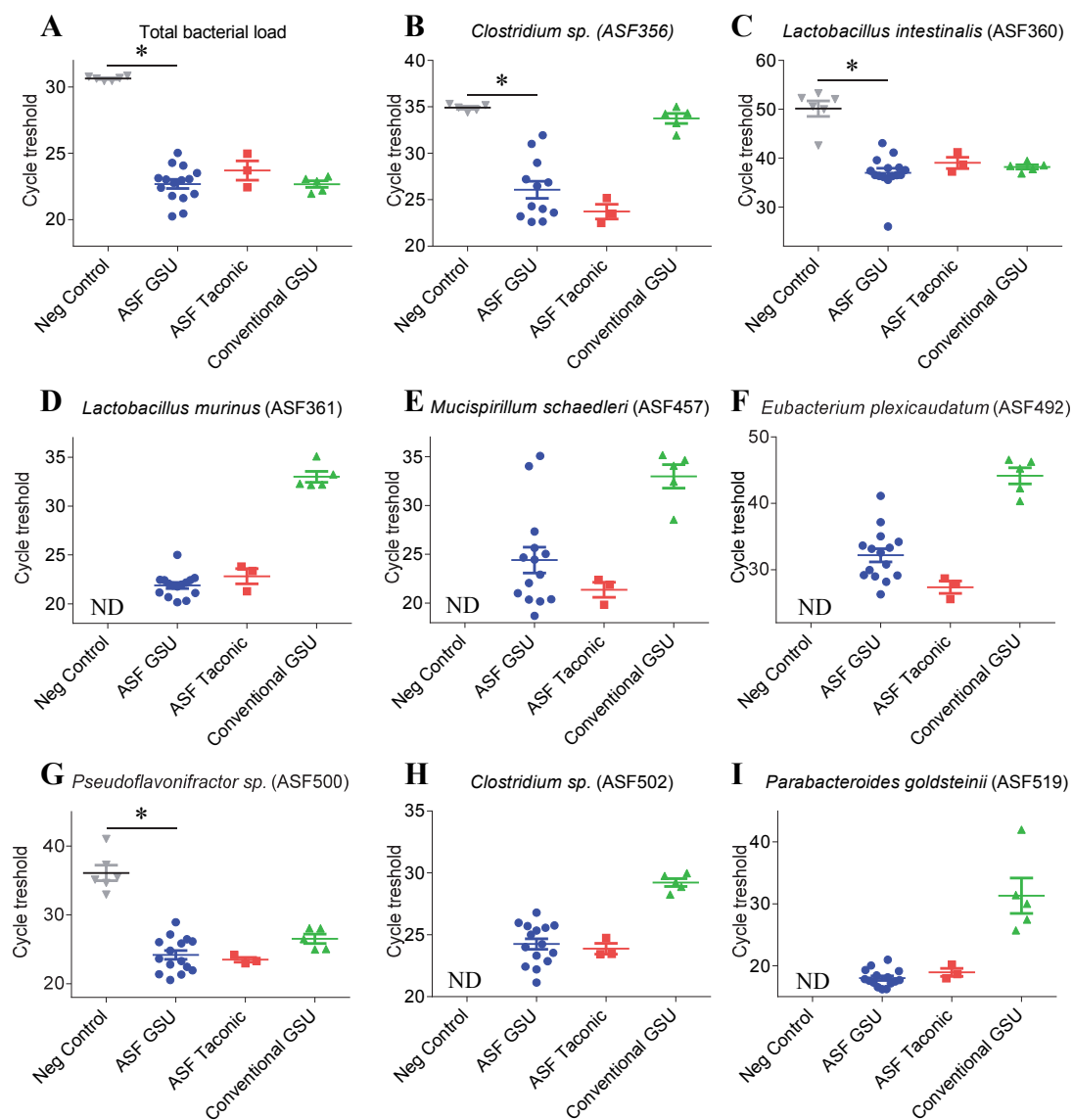

Supplement: S1 Fig — Feces were collected from conventional WT C57BL/6 and from WT C57BL/6 animals kept under ASF conditions at Taconic facility or at Georgia State University. A. Total bacterial load (515F-806R) cycle threshold values. B. Clostridium sp. (ASF 356) cycle threshold values. C. Lactobacillus intestinalis (ASF 360) cycle threshold values. D. Lactobacillus murinus (ASF 361) cycle threshold values. E. Mucispirillum shaedleri (ASF 457) cycle threshold values. F. Eubacterium plexicaudatum (ASF 492) cycle threshold values. G. Pseudoflavonifractor sp. (ASF 500) cycle threshold values. H. Clostridium sp. (ASF 502) cycle threshold values. I. Parabacteroides goldsteinii (ASF 519) cycle threshold values. n = 3–19. Significance was determined using t-test (* indicates p<0.05). (PDF) [file pone.0195310.s001.pdf]

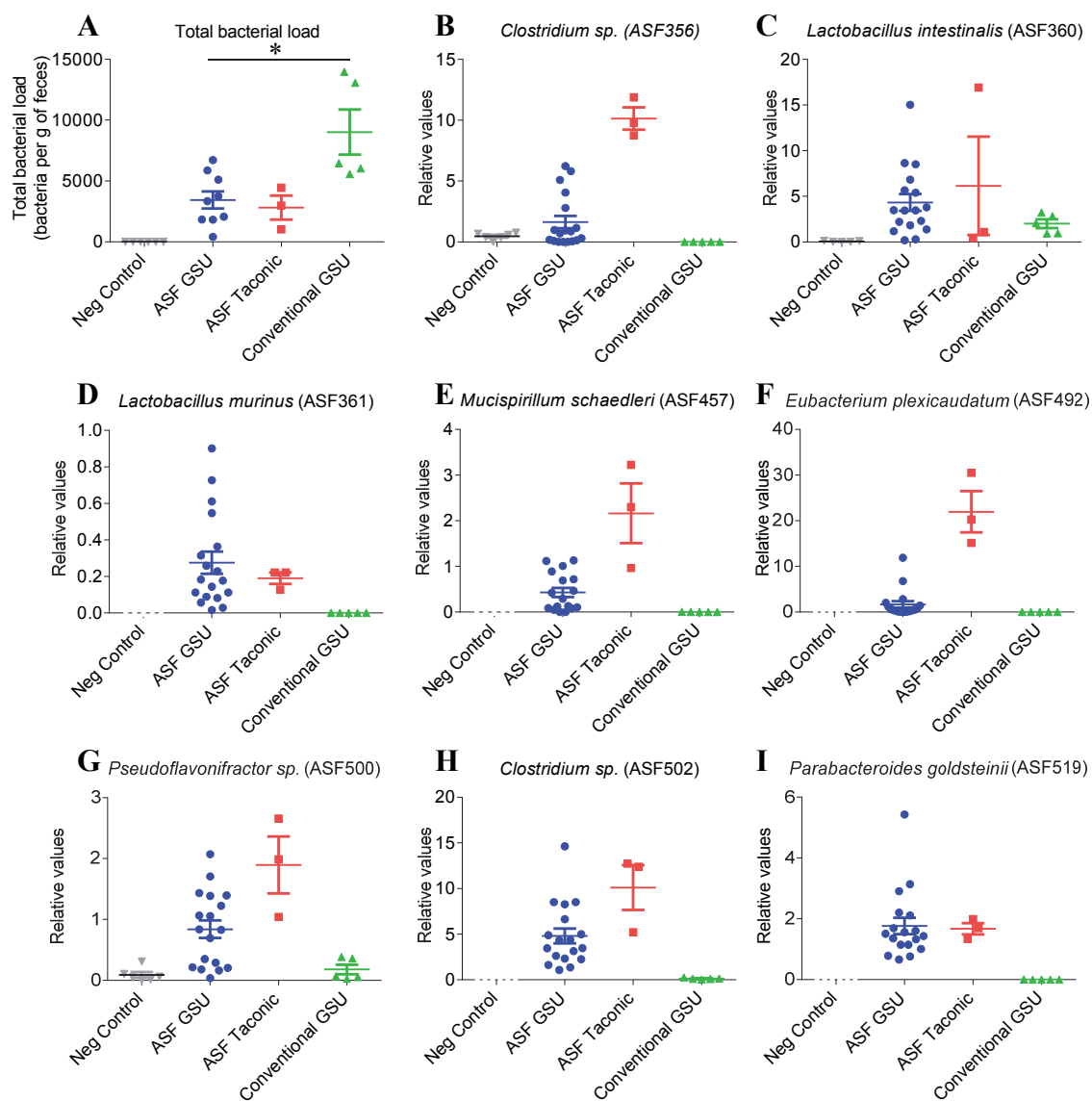

Supplement: S2 Fig — Feces were collected from conventional WT C57BL/6 and from WT C57BL/6 animals kept under ASF conditions at Taconic facility or at Georgia State University. A. Total bacterial load (515F-806R) express in bacteria per g of feces. B. Clostridium sp. (ASF 356) relative values, normalized with feces weight. C. Lactobacillus intestinalis (ASF 360) relative values, normalized with feces weight. D. Lactobacillus murinus (ASF 361) relative values, normalized with feces weight. E. Mucispirillum shaedleri (ASF 457) relative values, normalized with feces weight. F. Eubacterium plexicaudatum (ASF 492) relative values, normalized with feces weight. G. Pseudoflavonifractor sp. (ASF 500) relative values, normalized with feces weight. H. Clostridium sp. (ASF 502) relative values, normalized with feces weight. I. Parabacteroides goldsteinii (ASF 519) relative values, normalized with feces weight. n = 3–19. Significance was determined using t-test (* indicates p<0.05). (PDF) [file pone.0195310.s002.pdf]

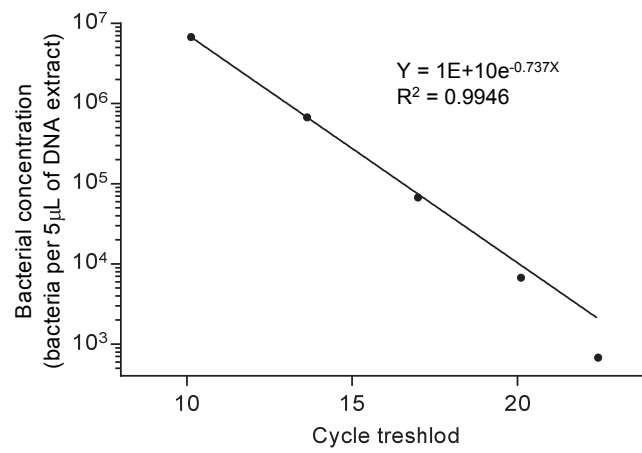

Supplement: S3 Fig — Bacterial DNA was extracted using QIAamp DNA Stool Mini Kit (Qiagen) from a serially diluted (1:10) overnight Escherichia coli culture from which we determined the exact bacterial concentration by plating bacterial culture on LB agar plate. Five μL of DNA was then subjected to quantitative PCR using QuantiFast SYBR Green PCR kit (Biorad) with universal 16S rRNA primers. Negative control = DNA extraction protocol was applied on water samples. Cycle threshold values vs bacterial concentration were plotted in order to determine the equation to use for bacterial density determination (Y = 1E+10e-0.737X). Non-linear (X is linear, Y is exponential) regression line was draft and R2 was determined. (PDF) [file pone.0195310.s003.pdf]

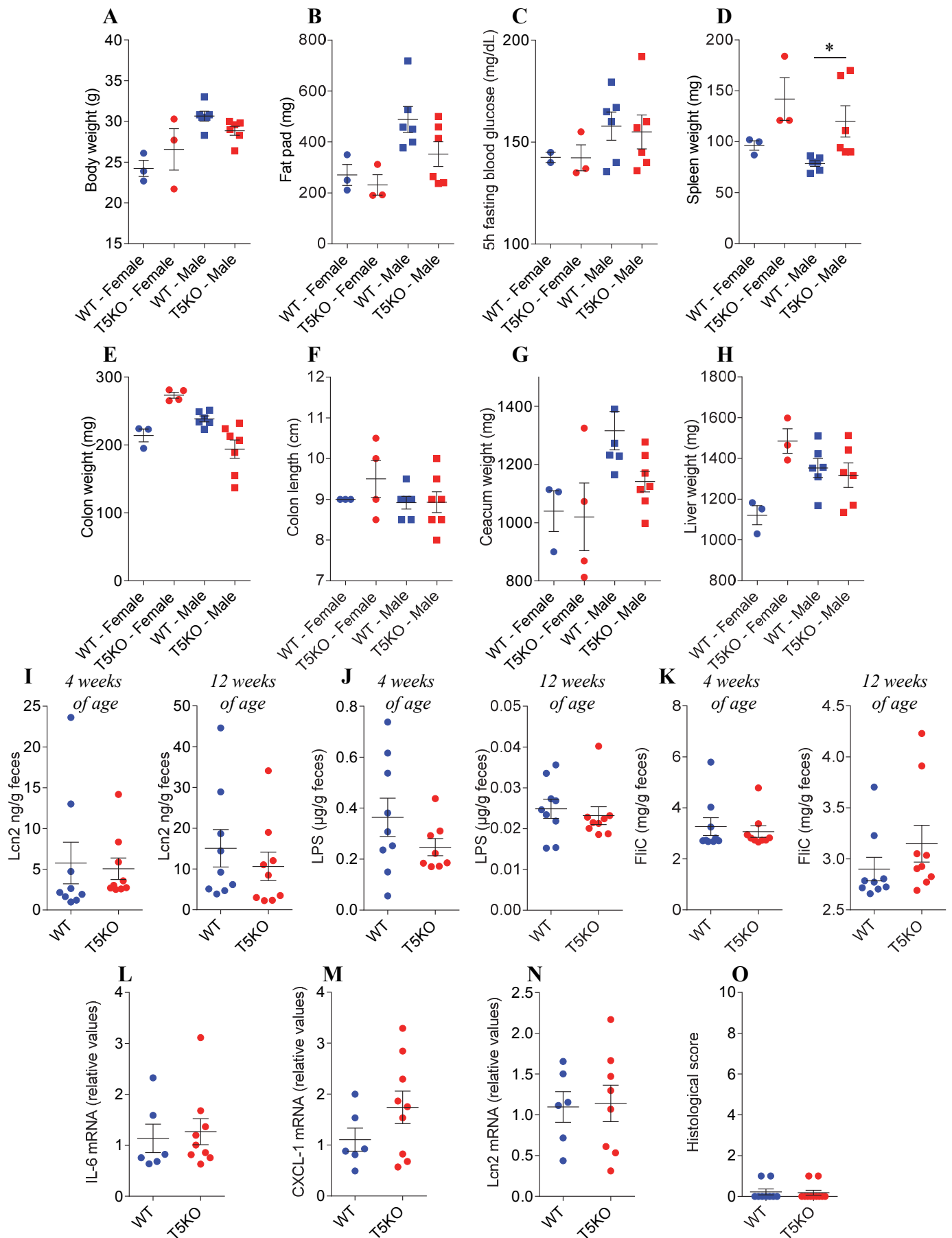

Chassaing et al., Figure S4.

Supplement: S4 Fig — WT and T5KO C57BL/6 mice, both males and females, were born from mice colonized with the Altered Schaedler Flora and maintained in isolators. At 12 weeks of age, mice were euthanized. A. Final body weight. B. Fat pad weight. C. 5 h fasting blood glucose concentration. D. Spleen weight. E. Colon weight. F. Colon length. G. Caecum weight. H. Liver weight. I. Fecal Lcn2 levels at week 4 and week 12 of age. J. Fecal lipopolysaccharide (LPS) levels at week 4 and week 12 of age. K. Fecal flagellin (FliC) levels at week 4 and week 12 of age. L-N. Colonic pro-inflammatory cytokine-encoding genes were quantified by qRT-PCR (L, IL-6; M, CXCL-1; N, Lcn2). O. Hematoxylin & eosin staining was performed on colonic sections and used for the determination of histopathological scores. Each dot represents one animals, bars represent means +/- S.E.M.. The data presented here are the same as Fig 1. (n = 3–6). Significance was determined using t-test (* indicates p<0.05). (PDF) [file pone.0195310.s004.pdf]

WT

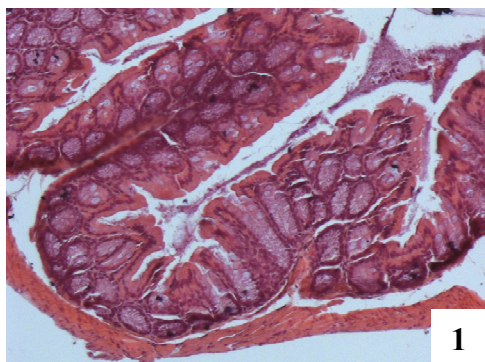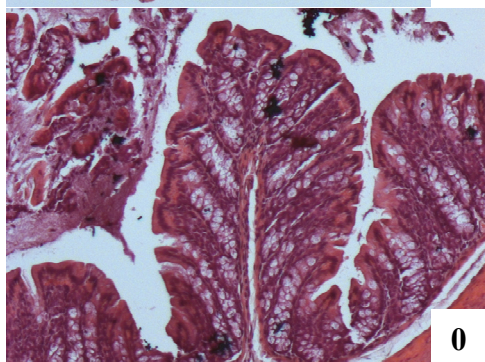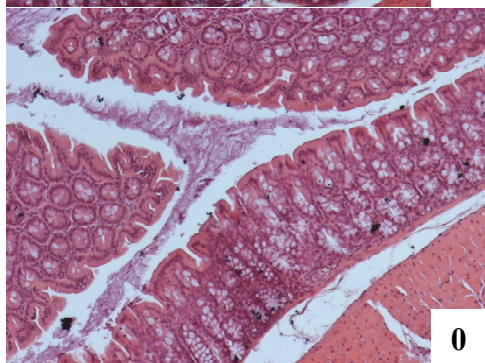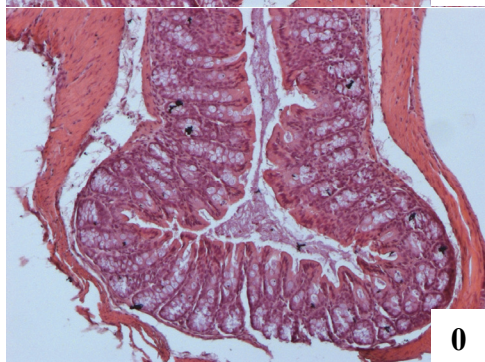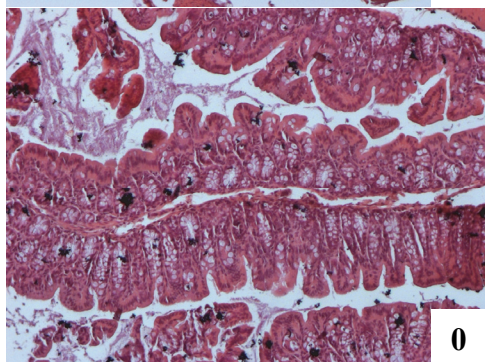

T5KO

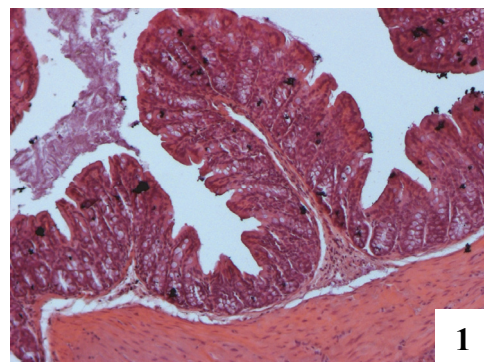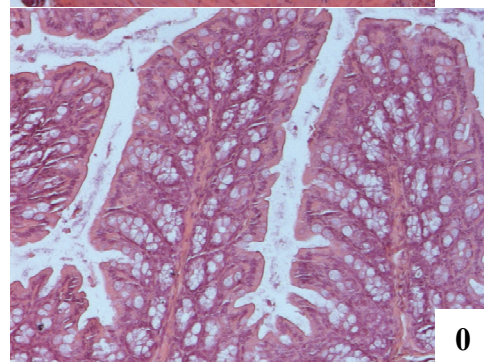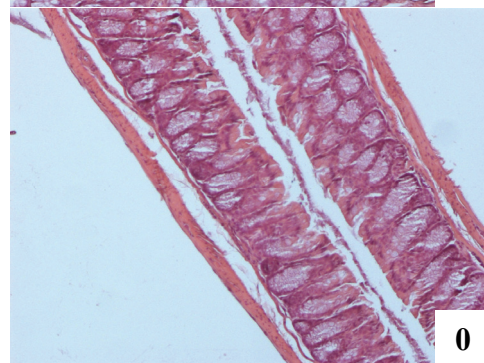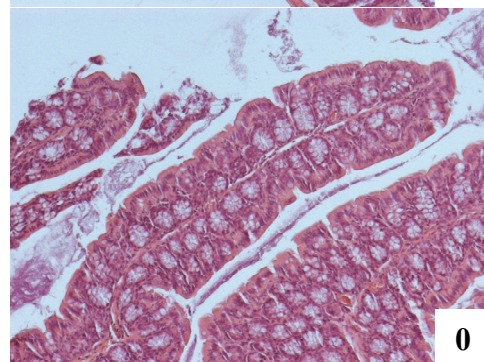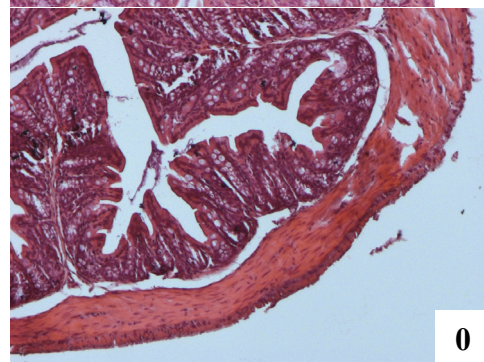

Supplement: S5 Fig — WT and T5KO C57BL/6 mice, both males and females, were born from mice colonized with the Altered Schaedler Flora and maintained in isolators. At 12 weeks of age, mice were euthanized. Hematoxylin & eosin staining was performed on colonic sections, and representative images were selected from 1–2 animals per cage. White boxes indicate individual histological score. (PDF) [file pone.0195310.s005.pdf]

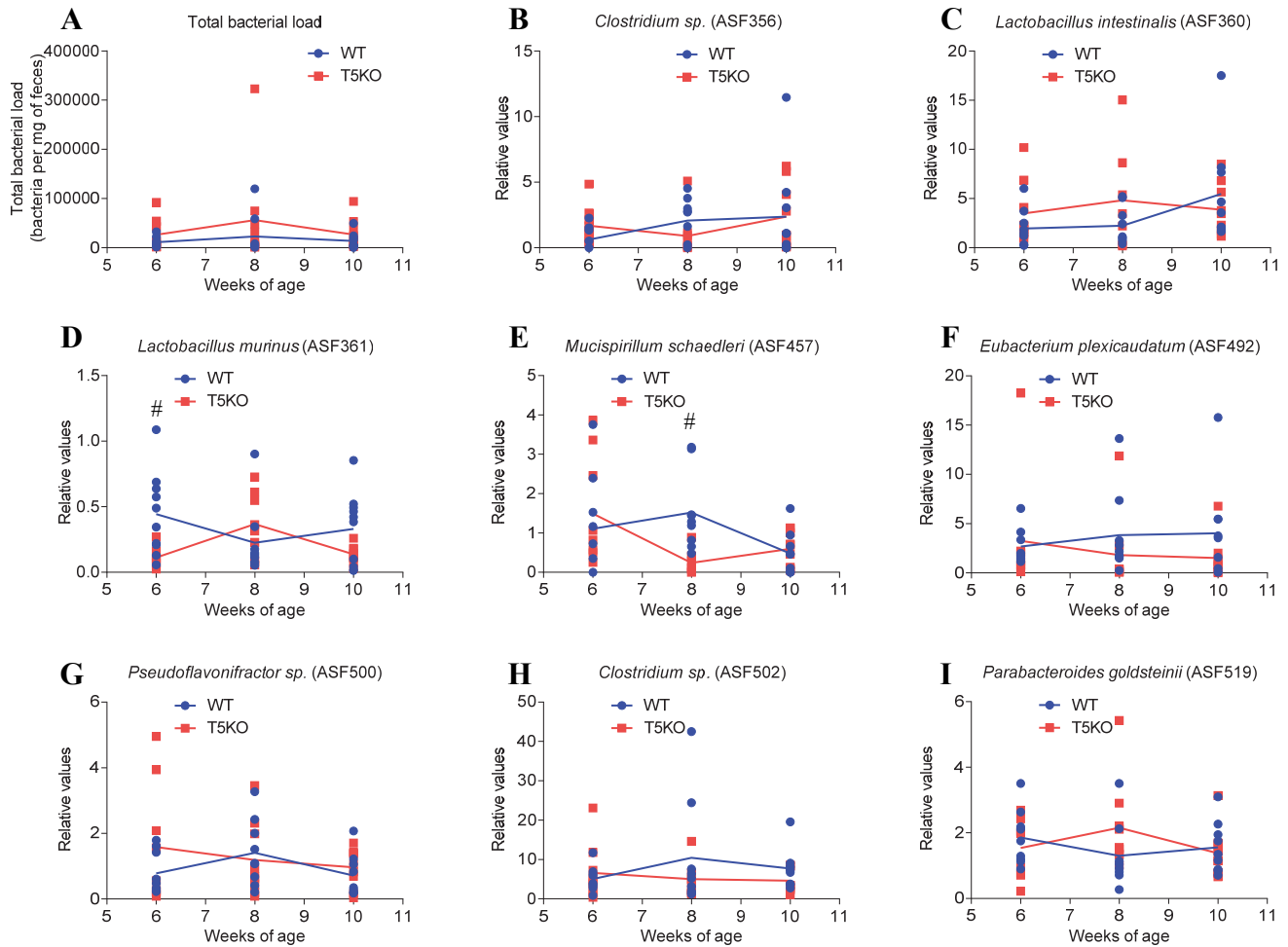

Supplement: S6 Fig — WT and T5KO mice, both males and females, were born from ASF-colonized mice and maintained in isolators. Feces were collected at weeks 6, 8 and 10 of age. A. Total bacterial load. B. Clostridium sp. (ASF 356) relative values. C. Lactobacillus intestinalis (ASF 360) relative values. D. Lactobacillus murinus (ASF 361) relative values. E. Mucispirillum shaedleri (ASF 457) relative values. F. Eubacterium plexicaudatum (ASF 492) relative values. G. Pseudoflavonifractor sp. (ASF 500) relative values. H. Clostridium sp. (ASF 502) relative values. I. Parabacteroides goldsteinii (ASF 519) relative values. Each dot represents one animals, bars represent mean. The data presented here are the same as Fig 2. n = 7–9. Significance was determined using two-way group ANOVA corrected for multiple comparisons with a Bonferroni test (# indicates statistical significance). (PDF) [file pone.0195310.s006.pdf]

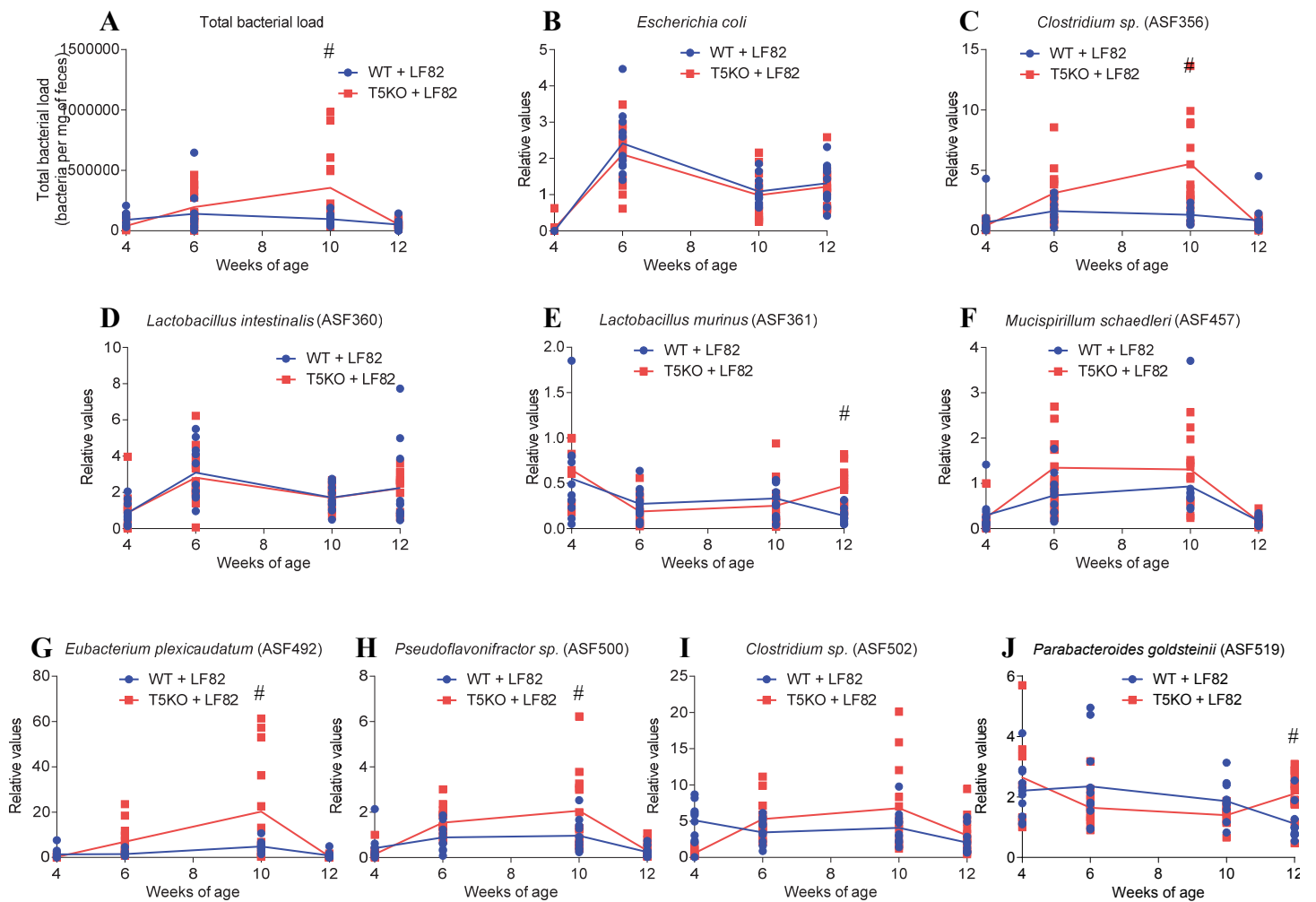

Chassaing et al., Figure S7.

Supplement: S7 Fig — Four-week old offspring of ASF-colonized WT and T5KO mice were removed from the isolator, placed in isolated ventilated cages and inoculated with AIEC reference strain LF82 placed in drinking water for two weeks, followed by return to autoclaved water. Feces were collected at weeks 4, 6, 10 and 12 of age. A. Total bacterial load. B. E. coli relative values. C. Clostridium sp. (ASF 356) relative values. D. Lactobacillus intestinalis (ASF 360) relative values. E. Lactobacillus murinus (ASF 361) relative values. F. Mucispirillum shaedleri (ASF 457) relative values. G. Eubacterium plexicaudatum (ASF 492) relative values. H. Pseudoflavonifractor sp. (ASF 500) relative values. I. Clostridium sp. (ASF 502) relative values. J. Parabacteroides goldsteinii (ASF 519) relative values. Each dot represents one animals, bars represent mean. The data presented here are the same as Fig 3. n = 8–13. Significance was determined using two-way group ANOVA corrected for multiple comparisons with a Bonferroni test (# indicates statistical significance). (PDF) [file pone.0195310.s007.pdf]

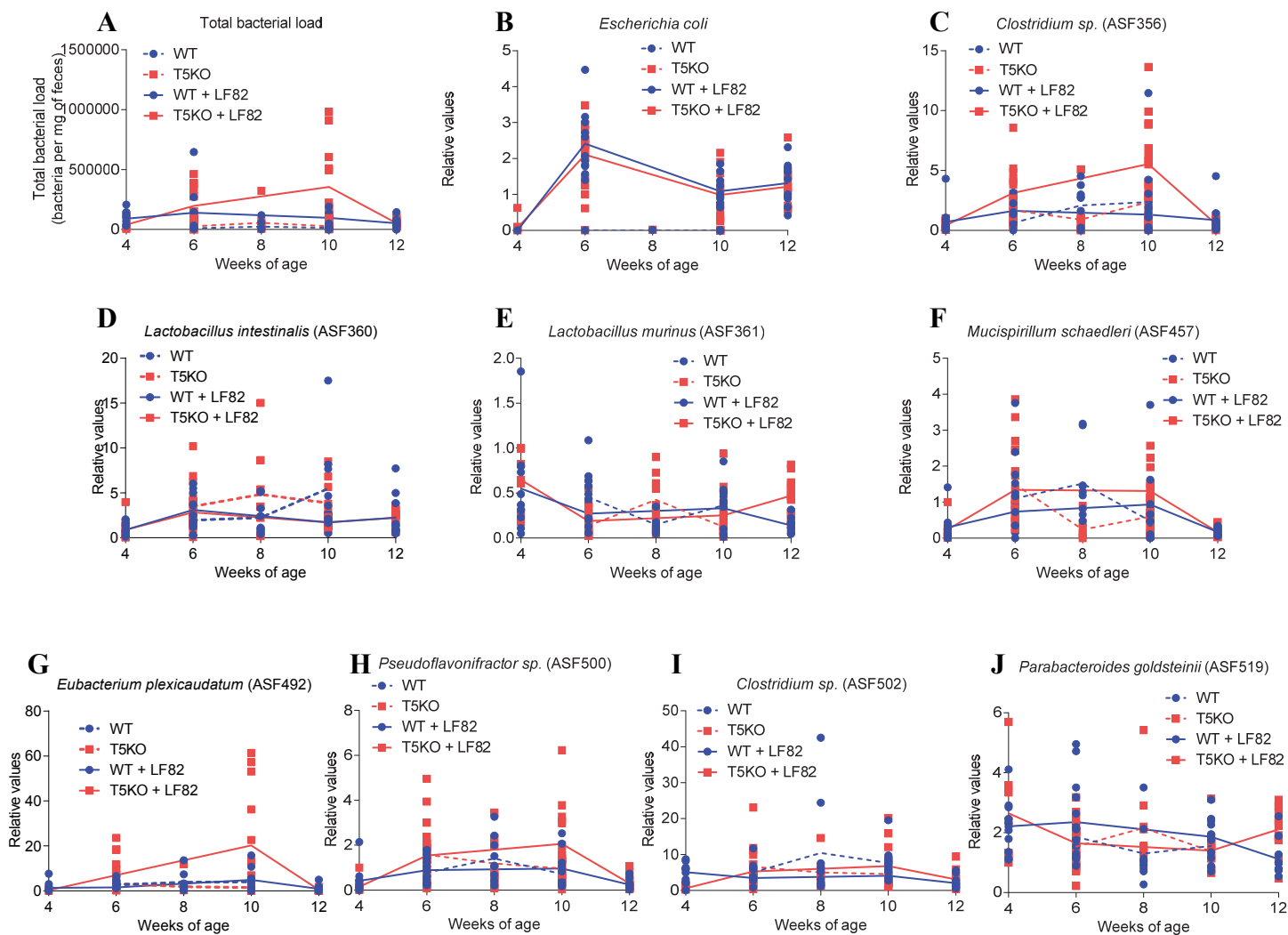

Supplement: S8 Fig — Four-week old offspring of ASF-colonized WT and T5KO mice were remove from the isolator, placed in isolated ventilated cages and inoculated with AIEC reference strain LF82 placed in drinking water for two weeks, followed by return to autoclaved water. Feces were collected at weeks 4, 6, 8, 10 and 12 of age. A. Total bacterial load. B. E. coli relative values. C. Clostridium sp. (ASF 356) relative values. D. Lactobacillus intestinalis (ASF 360) relative values. E. Lactobacillus murinus (ASF 361) relative values. F. Mucispirillum shaedleri (ASF 457) relative values. G. Eubacterium plexicaudatum (ASF 492) relative values. H. Pseudoflavonifractor sp. (ASF 500) relative values. I. Clostridium sp. (ASF 502) relative values. J. Parabacteroides goldsteinii (ASF 519) relative values. Each dot represents one animals, bars represent mean. The data presented here are the same as Fig 4. n = 8–13. (PDF) [file pone.0195310.s008.pdf]

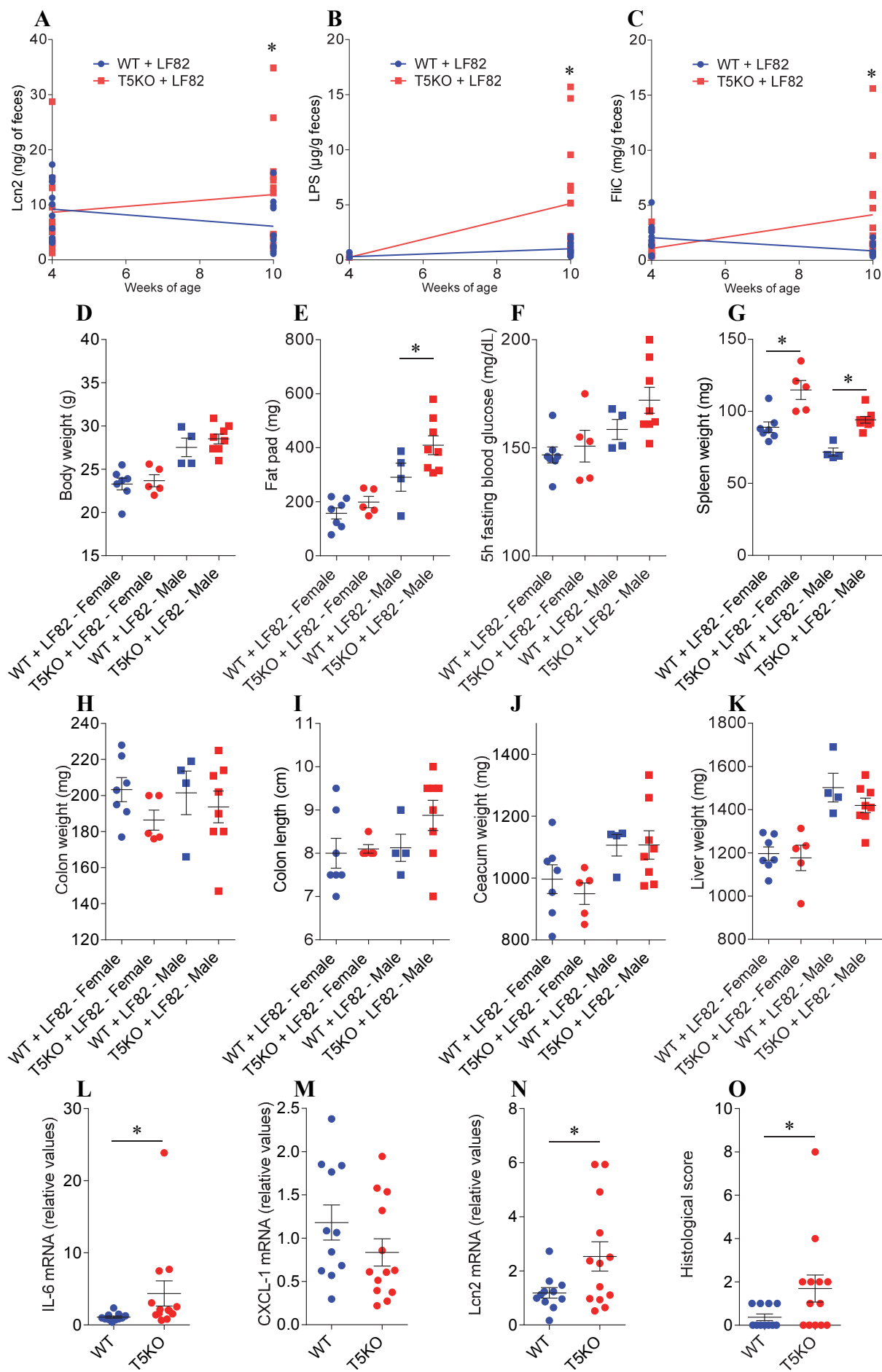

Chassaing et al., Figure S9.

Supplement: S9 Fig — Four-week old offspring of ASF-colonized WT and T5KO mice were removed from the isolator, placed in isolated ventilated cages and inoculated with AIEC reference strain LF82 placed in drinking water for two weeks, followed by return to autoclaved water. At 12 weeks of age, animals were removed from this isolator and euthanized. A. Fecal Lcn2 levels at week 4 and week 10 of age. B. Fecal lipopolysaccharide (LPS) levels at week 4 and week 10 of age. C. Fecal flagellin (FliC) levels at week 4 and week 10 of age. D. Final body weight. E. Fat pad weight. F. 5 h fasting blood glucose concentration. G. Spleen weight. H. Colon weight. I. Colon length. J. Caecum weight. K. Liver weight. L-N. Colonic pro-inflammatory cytokine-encoding genes were quantified by qRT-PCR (L, IL-6; M, CXCL-1; N, Lcn2). O. Hematoxylin & eosin staining was performed on colonic sections and used for the determination of histopathological scores. Each dot represents one animals, bars represent means +/- S.E.M.. The data presented here are the same as Fig 5. (n = 3–6). Data in A, B and C combine both male and female animals. Significance was determined using t-test (* indicates p<0.05). (PDF) [file pone.0195310.s009.pdf]

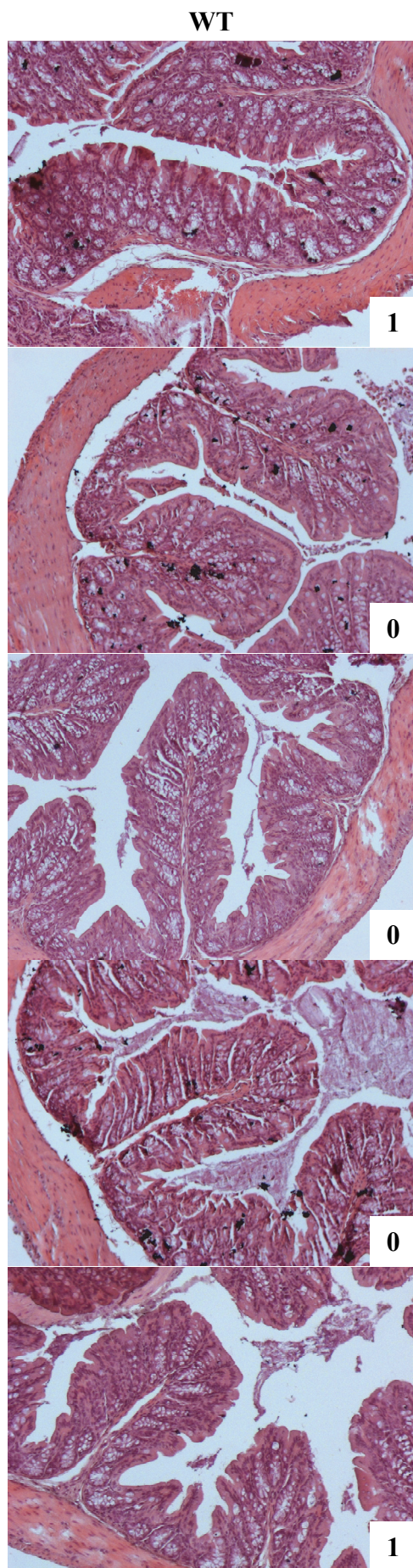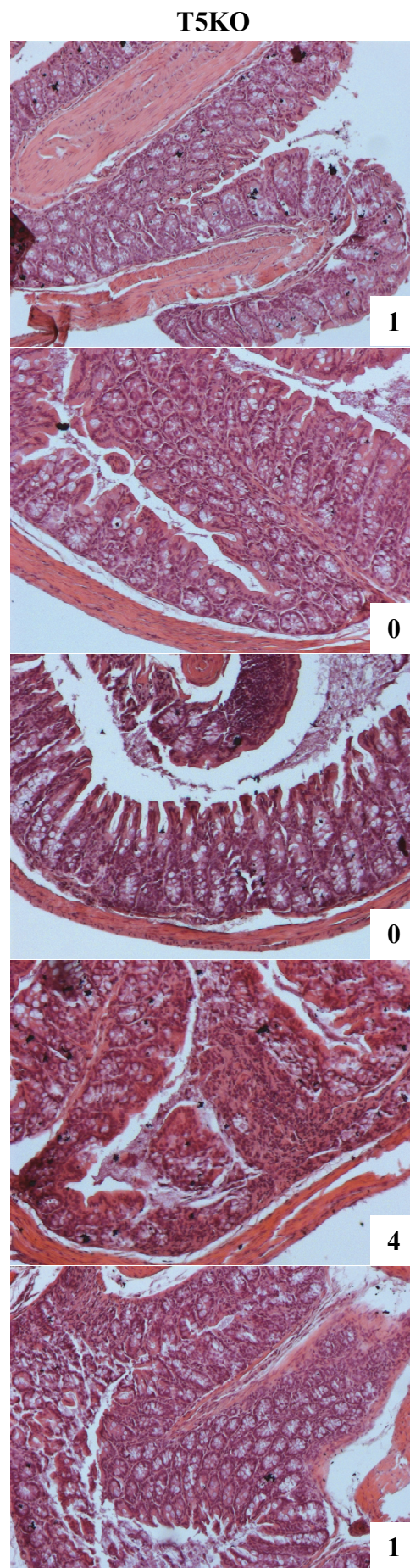

Chassaing et al., Figure S10.

Supplement: S10 Fig — Four-week old offspring of ASF-colonized WT and T5KO mice were removed from the isolator, placed in isolated ventilated cages and inoculated with AIEC reference strain LF82 placed in drinking water for two weeks, followed by return to autoclaved water. At 12 weeks of age, animals were removed from this isolator and euthanized. Hematoxylin & eosin staining was performed on colonic sections, and representative images were selected from 1–2 animals per cage. White boxes indicate individual histological score. (PDF) [file pone.0195310.s010.pdf]

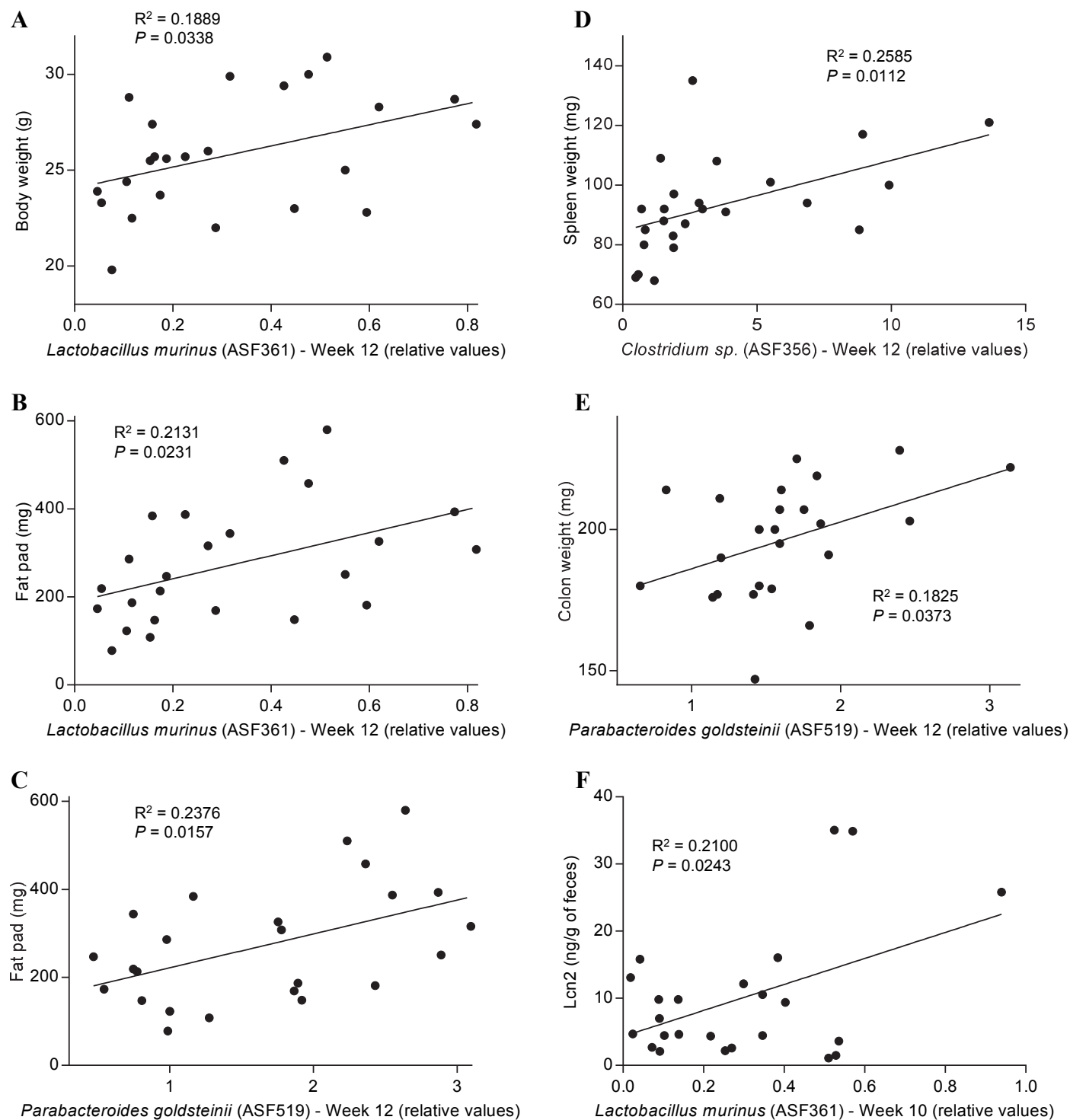

Chassaing et al., Figure S11.

Supplement: S11 Fig — Four-week old offspring of ASF-colonized WT and T5KO mice were removed from the isolator, placed in isolated ventilated cages and inoculated with AIEC reference strain LF82 placed in drinking water for two weeks, followed by return to autoclaved water. A. Relative values of Lactobacillus murinus (ASF 361) at week 12 and final body weights were plotted in X and Y axis, respectively. B. Relative values of Lactobacillus murinus (ASF 361) at week 12 and fat pad weights were plotted in X and Y axis, respectively. C. Relative values of Parabacteroides goldsteinii (ASF 519) at week 12 and fat pad weights were plotted in X and Y axis, respectively. D. Relative values of Clostridium sp. (ASF 356) at week 12 and spleen weights were plotted in X and Y axis, respectively. E. Relative values of Parabacteroides goldsteinii (ASF 519) at week 12 and colon weights were plotted in X and Y axis, respectively. F. Relative values of Lactobacillus murinus (ASF 361) at week 10 and final fecal Lcn2 levels were plotted in X and Y axis, respectively. Linear regression lines were drafted and R2 were determined. (n = 11). (PDF) [file pone.0195310.s011.pdf]
